# Dapagliflozin and atrial fibrillation in heart failure with reduced ejection fraction: insights from DAPA‐HF

**DOI:** 10.1002/ejhf.2381

**Published:** 2021-11-24

**Authors:** Jawad H. Butt, Kieran F. Docherty, Pardeep S. Jhund, Rudolf A. de Boer, Michael Böhm, Akshay S. Desai, Jonathan G. Howlett, Silvio E. Inzucchi, Mikhail N. Kosiborod, Felipe A. Martinez, Jose C. Nicolau, Mark C. Petrie, Piotr Ponikowski, Olof Bengtsson, Anna Maria Langkilde, Morten Schou, Mikaela Sjöstrand, Scott D. Solomon, Marc S. Sabatine, John J.V. McMurray, Lars Køber

**Affiliations:** ^1^ British Heart Foundation Cardiovascular Research Centre University of Glasgow Glasgow UK; ^2^ Department of Cardiology Rigshospitalet Copenhagen University Hospital Copenhagen Denmark; ^3^ Department of Cardiology University Medical Center and University of Groningen Groningen The Netherlands; ^4^ Department of Internal Medicine III, Saarland University Hospital Saarland University Homburg Germany; ^5^ Division of Cardiovascular Medicine Brigham and Women's Hospital Boston MA USA; ^6^ Cumming School of Medicine and Libin Cardiovascular Institute University of Calgary Calgary Canada; ^7^ Section of Endocrinology Yale School of Medicine New Haven CT USA; ^8^ Saint Luke's Mid America Heart Institute, University of Missouri, Kansas City; The George Institute for Global Health University of New South Wales Sydney Australia; ^9^ Universidad Nacional de Córdoba Córdoba Argentina; ^10^ Instituto do Coracao (InCor), Hospital das Clínicas Faculdade de Medicina Universidade de São Paulo São Paulo Brazil; ^11^ Center for Heart Diseases University Hospital, Wroclaw Medical University Wrocław Poland; ^12^ Late Stage Development, Cardiovascular, Renal and Metabolism BioPharmaceuticals R&D, AstraZeneca Gothenburg Sweden; ^13^ Department of Cardiology Herlev‐Gentofte University Hospital Herlev Denmark; ^14^ TIMI Study Group, Division of Cardiovascular Medicine Brigham and Women's Hospital Boston MA USA

**Keywords:** Heart failure, Dapagliflozin, Atrial fibrillation, Randomized trial

## Abstract

**Aims:**

Among patients with heart failure (HF) and reduced ejection fraction (HFrEF), those with atrial fibrillation (AF) may respond differently to certain treatments than patients without AF. We investigated the efficacy and safety of dapagliflozin in patients with HFrEF with and without AF in the Dapagliflozin And Prevention of Adverse‐outcomes in Heart Failure trial (DAPA‐HF). We also examined the effect of dapagliflozin on new‐onset AF.

**Methods and results:**

The primary outcome was the composite of an episode of worsening HF (HF hospitalization or urgent HF visit requiring intravenous therapy) or cardiovascular death. Of the 4744 patients randomized, 1910 (40.3%) had ‘any AF’ (history of AF or AF on enrolment electrocardiogram). Compared with placebo, dapagliflozin reduced the risk of worsening HF or cardiovascular death to a similar extent in patients with and without any AF [hazard ratio (HR) 0.75, 95% confidence interval (CI) 0.62–0.92 and 0.74, 95% CI 0.62–0.88, respectively; *p* for interaction = 0.88]. Consistent benefits were observed for the components of the primary outcome, all‐cause mortality, and improvement of Kansas City Cardiomyopathy Questionnaire total symptom score. Among patients without AF at baseline, dapagliflozin did not significantly reduce the risk of new‐onset AF compared with placebo (HR 0.86, 95% CI 0.60–1.22). However, patients with new‐onset AF had a 5 to 6‐fold higher risk of adverse outcomes when compared to those without incident AF.

**Conclusions:**

Dapagliflozin, compared with placebo, reduced the risk of worsening HF events, cardiovascular death, and all‐cause death, and improved symptoms, in patients with and without AF. Dapagliflozin did not reduce the risk of new‐onset AF.

## Graphical abstract

Dapagliflozin reduced the risk of worsening heart failure events and death, and improved symptoms, irrespective of atrial fibrillation (AF) status. However, dapagliflozin did not reduce the risk of new‐onset AF. (*A*) Effects of dapagliflozin compared with placebo on clinical outcomes according AF status at baseline. (*B*) Cumulative incidence of new‐onset AF in DAPA‐HF according to randomized treatment assignment in patients without AF at baseline (neither a history or AF on the baseline electrocardiogram).

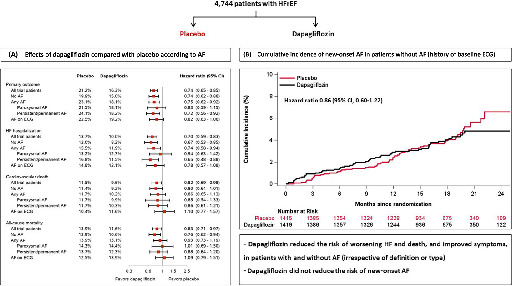

## Introduction

Atrial fibrillation (AF) and heart failure (HF) with reduced ejection fraction (HFrEF) often coexist, and each increases the likelihood and complicates the course and treatment of the other.^1^, ^2^ Patients with HFrEF and AF are generally older, have a greater symptom burden, lower quality of life, and more comorbidity than those without AF.^3^, ^4^, ^5^, ^6^ Patients with AF are also at higher risk of adverse outcomes, including HF hospitalization and death, although AF may be a marker of more advanced HF, rather than an independent prognostic risk factor.^1^, ^3^, ^4^, ^5^, ^6^, ^7^, ^8^, ^9^, ^10^, ^11^, ^12^ Patients with paroxysmal AF may be at higher risk than those with persistent or permanent AF.^3^

Sodium–glucose co‐transporter 2 (SGLT2) inhibitors, originally developed as glucose‐lowering agents for type 2 diabetes, are now a valuable new treatment for HFrEF, including in patients without diabetes.^13^, ^14^ Their effects in HFrEF patients with AF is of interest, from two perspectives. First, some treatments cannot be used in AF (e.g. ivabradine) and the effectiveness of others may be modified by the presence of AF (e.g. beta‐blockers, cardiac resynchronization therapy and, most recently, omecamtiv mecarbil).^15^, ^16^, ^17^, ^18^ Second, new‐onset AF is associated with a high risk of adverse outcomes, including HF hospitalization, stroke, and all‐cause death in patients with HFrEF,^3^, ^4^, ^5^, ^11^, ^19^, ^20^ and some guideline‐recommended therapies for HFrEF reduce the incidence of new‐onset AF.^6^, ^15^, ^21^, ^22^, ^23^ Recently, a *post hoc* analysis of the Dapagliflozin Effect on Cardiovascular Events–Thrombolysis in Myocardial Infarction 58 trial (DECLARE‐TIMI 58) also suggested that dapagliflozin decreased the risk of AF events in patients with type 2 diabetes, irrespective of a history of HF.^24^ It is therefore important to not only evaluate the efficacy and safety of dapagliflozin in HFrEF patients with and without AF, but also to investigate whether this drug reduces the incidence of new‐onset AF in a well‐defined HFrEF population.

We examined both these questions in the Dapagliflozin And Prevention of Adverse‐outcomes in Heart Failure trial (DAPA‐HF), which demonstrated that dapagliflozin, compared with placebo, reduced the risk of worsening HF events and death, and improved symptoms, when added to standard therapy in 4744 patients with HFrEF.^13^

## Methods

DAPA‐HF was a randomized, double‐blind, placebo‐controlled trial in patients with HFrEF, evaluating the efficacy and safety of dapagliflozin 10 mg once daily compared with matching placebo, added to standard care. The design, baseline characteristics, and primary results of the trial are published.^13^, ^25^, ^26^ The Ethics Committee of each of the 410 participating institutions in 20 countries approved the protocol, and all patients gave written informed consent. The corresponding author had full access to the trial data and takes responsibility for its integrity and the data analysis.

### Study patients

Patients aged 18 years or older with a diagnosis of HF for at least 2 months were eligible if they were in New York Heart Association (NYHA) functional class II–IV, had a left ventricular ejection fraction (LVEF) of ≤40%, were optimally treated with pharmacological and device therapy, and had an N‐terminal pro‐B‐type natriuretic peptide (NT‐proBNP) concentration ≥600 pg/ml [≥400 pg/ml if hospitalized for HF within the previous 12 months; ≥900 pg/ml if AF on the electrocardiogram (ECG) at enrolment, irrespective of history of HF hospitalization]. Key exclusion criteria included symptoms of hypotension or systolic blood pressure < 95 mmHg, estimated glomerular filtration rate (eGFR) <30 ml/min/1.73 m^2^ or rapidly declining renal function, type 1 diabetes, and other conditions likely to prevent patient participation in the trial or greatly limit life expectancy. A complete list of exclusion criteria is provided in the design paper.^25^ After randomization, follow‐up visits were scheduled at 14, 60, and 120 days and then every 4 months thereafter until end of follow‐up (withdrawal of consent, death, or 5 June 2019).

### Atrial fibrillation

Data about history of AF were collected on the trial case report forms (CRF). Investigators were first asked whether patients had a history of AF any time before enrolment. If investigators answered yes, they were then asked to specify the type of AF according to the following options: paroxysmal [intermittent (lasting at least 30 s), self‐terminating AF (lasting for a maximum of 1 week)], persistent (non‐self‐terminating AF with a duration of >1 week and/or required cardioversion), and permanent (non‐self‐terminating, long‐standing AF in which cardioversion has failed or not attempted). Data on heart rhythm on the ECG at enrolment were also collected on the CRF, and investigators were asked to specify the heart rhythm from the following options: sinus rhythm, AF, atrial flutter, paced rhythm, and other (specify).

New‐onset (incident) AF was a clinical endpoint in patients without any AF at baseline (i.e. no history of AF and no AF on ECG at enrolment), and data were collected from a specific CRF. Investigators were asked to specify the date of diagnosis, symptoms (symptomatic or asymptomatic), type of AF (paroxysmal, persistent, or permanent), and treatment [rate control therapy, antiarrhythmic drugs, invasive antiarrhythmic therapy (percutaneous/surgical ablation or pacemaker insertion to facilitate rhythm control), electric cardioversion, antiplatelet therapy, or anticoagulant therapy].

### Comparison groups of interest

In the present study, we made the following comparisons based on AF status:

1. Patients without any AF (no history of AF and no AF on ECG) vs. any AF (a history of AF or AF on ECG at enrolment). ‘Atrial fibrillation’ in these analyses included AF or atrial flutter as in previous studies.^3^, ^4^, ^5^, ^6^, ^7^, ^8^, ^9^, ^10^, ^11^, ^12^
2. Patients without any AF (no history of AF and no AF on ECG) vs. AF on ECG at enrolment (irrespective of history of AF).
3. Patients without any AF (no history of AF and no AF on ECG) vs. paroxysmal AF (history of paroxysmal AF or AF on ECG without a history of AF) vs. persistent/permanent AF (history of persistent/permanent AF).

### Trial outcomes

The primary outcome in DAPA‐HF was the composite of worsening HF (HF hospitalization or an urgent visit for worsening HF and administration of intravenous treatment for HF) or cardiovascular death. The secondary outcomes in the trial were the occurrence of HF hospitalization or cardiovascular death (we also examined the components of this composite); total HF hospitalizations (first and repeat) or cardiovascular death; change from baseline to 8 months in the Kansas City Cardiomyopathy Questionnaire total symptom score (KCCQ‐TSS); and death from any cause. Pre‐specified safety analyses included serious adverse events, adverse events leading to discontinuation of trial treatment, and adverse events of interest, including volume depletion, renal adverse events, bone fracture, amputation, major hypoglycaemia, and diabetic ketoacidosis. Safety analyses were performed in patients who had undergone enrolment and received at least one dose of either dapagliflozin or placebo (a total of eight randomized patients were excluded from the safety analysis).

In the present study, we investigated the association between AF status and the risk of the composite of worsening HF or cardiovascular death; HF hospitalization or cardiovascular death (as well as the components of this composite); all‐cause death; and stroke. We also evaluated the efficacy and safety of dapagliflozin, compared with placebo, according to AF status. Finally, we examined the risk of incident AF with dapagliflozin, compared with placebo, in patients without any AF (no history of AF and no AF on ECG at enrolment).

Atrial fibrillation on enrolment ECG was a pre‐specified subgroup analysis, but the assessment of secondary clinical outcomes by AF (irrespective of definition) was done *post hoc*. The incidence of a new diagnosis of AF in patients without AF at baseline was a pre‐specified exploratory endpoint.

### Statistical analyses

Baseline characteristics were summarized as frequencies with percentages, means with standard deviation (SD), or medians with interquartile ranges. Differences in baseline characteristics were tested using the chi‐square test for categorical variables and the Wilcoxon test and two‐sample *t*‐test for non‐normal and normally distributed continuous variables, respectively.

Time‐to‐event data, regardless of treatment allocation, were evaluated using Cox proportional‐hazards models, stratified according to diabetes mellitus status, with a history of HF hospitalization and treatment‐group assignment as fixed‐effect factors to calculate hazard ratios (HR) with 95% confidence intervals (CIs). In addition, adjusted HRs from models including age, sex, geographical region, heart rate, systolic blood pressure, body mass index, HF aetiology, LVEF, NYHA functional class, NT‐proBNP, eGFR, hypertension, chronic obstructive pulmonary disease, and prior stroke/transient ischaemic attack were reported. Adjusted HRs, but without adjustment for NT‐proBNP, were also reported. To assess the prognostic significance of new‐onset AF during follow‐up among patients without any AF at enrolment, AF was included as a time‐dependent variable in a Cox proportional‐hazards model, adjusted for the variables previously mentioned. Patients who developed new‐onset AF were removed from the no‐AF subgroup and classified as new‐onset AF from the date of new‐onset AF and onward.

To compare the effects of dapagliflozin vs. placebo on the clinical outcomes, time‐to‐event data were evaluated with cause‐specific Cox proportional‐hazards models, stratified according to diabetes mellitus status, with a history of HF hospitalization and treatment‐group assignment as fixed‐effect factors. Total, including recurrent events were evaluated with semiparametric proportional‐rates models.^27^ The difference between treatment groups in the change in KCCQ‐TSS from baseline to 8 months in surviving patients was analysed using two‐sample *t*‐test. Responder analyses examining proportions of patients with a deterioration (decrease of ≥5 points) and improvement (increase of ≥5 points) in KCCQ scores at 8 months were conducted with the treatment effect expressed as an odds ratio with 95% CI using methods previously described.^28^

To compare the effect of dapagliflozin vs. placebo on new‐onset AF, time‐to‐event data were evaluated with the Aalen–Johansen estimator (taken the competing risk of death into account) and a cause‐specific Cox proportional‐hazards model, stratified according to diabetes mellitus status, with a history of HF hospitalization and treatment‐group assignment as fixed‐effect factors. A Fine–Gray competing risk analysis was also performed, with death from any cause considered a competing risk.

All analyses were conducted using SAS version 9.4 (SAS Institute, Cary, NC, USA) and STATA version 16.1 (Stata Corp., College Station, TX, USA). A *p*‐value of 0.05 was considered statistically significant.

## Results

### Patients characteristics

#### Any atrial fibrillation (history of AF or AF on baseline electrocardiogram)

Of the 4744 patients randomized, 1910 (40.3%) had ‘any AF’, i.e. history of AF or AF on ECG at enrolment. Baseline characteristics according to AF status are presented in *Table* *1*. Compared to patients without AF, those with AF were older, more often male and white (and less often Asian), more likely to have hypertension and chronic obstructive pulmonary disease, and had a higher CHA_2_DS_2_‐VASc score, body mass index, heart rate, and baseline NT‐proBNP, and lower eGFR. They also had a longer duration of HF and were less likely to have an ischaemic aetiology or prior myocardial infarction. Patients with AF had a higher LVEF, but worse NYHA functional class and KCCQ‐TSS than patients without AF. Regarding background HF therapy, patients with AF were less frequently treated with guideline‐recommended medical therapy, but they were more likely to have a defibrillating device. Overall, 84% of patients with AF were treated with an oral anticoagulant.

**Table 1:** Baseline characteristics of the study population according to atrial fibrillation (history or baseline electrocardiogram)

	No AF (*n* = 2834)	Any AF (*n* = 1910)	*p*‐value
Age (years), mean (SD)	64.3 (11.2)	69.3 (9.6)	<0.001
Sex, *n* (%)			<0.001
Female	713 (25.2)	396 (20.7)	
Male	2121 (74.8)	1514 (79.3)	
Race, *n* (%)			<0.001
Asian	806 (28.4)	310 (16.2)	
Black	156 (5.5)	70 (3.7)	
White	1825 (64.4)	1508 (79.0)	
Other	47 (1.7)	22 (1.2)	
Geographic region, *n* (%)			<0.001
Asia/Pacific	791 (27.9)	305 (16.0)	
Europe	1085 (38.3)	1069 (56.0)	
North America	391 (13.8)	286 (15.0)	
South America	567 (20.0)	250 (13.1)	
Physiologic measures			
Systolic blood pressure (mmHg), mean (SD)	121.9 (16.8)	121.7 (15.7)	0.58
Heart rate (bpm), mean (SD)	70.7 (10.7)	72.7 (12.9)	<0.001
BMI (kg/m^2^), mean (SD)	27.7 (5.9)	28.8 (6.0)	<0.001
Creatinine (µmol/L), mean (SD)	101.7 (30.6)	108.6 (29.7)	<0.001
Glycated haemoglobin, median (IQR)	6.1 (5.7–7.0)	6.1 (5.7–6.7)	0.05
eGFR (ml/min/1.73 m^2^), mean (SD)	68.5 (20.1)	61.7 (17.6)	<0.001
eGFR (ml/min/1.73 m^2^), *n* (%)			<0.001
<60	1001 (35.3)	925 (48.4)	
≥60	1831 (64.7)	985 (51.6)	
NT‐proBNP, median (IQR)	1242 (742–2325)	1.795 (1106–3041)	<0.001
Main cause of HF, *n* (%)			<0.001
Ischaemic	1723 (60.8)	951 (49.8)	
Non‐ischaemic	890 (31.4)	797 (41.7)	
Unknown	221 (7.8)	162 (8.5)	
Duration of HF, *n* (%)			<0.001
0–3 months	94 (3.3)	56 (2.9)	
3–6 months	260 (9.2)	133 (7.0)	
6–12 months	376 (13.3)	179 (9.4)	
1–2 years	450 (15.9)	236 (12.4)	
2–5 years	652 (23.0)	453 (23.7)	
>5 years	1002 (35.4)	853 (44.7)	
LVEF, mean (SD)	30.5 (6.8)	31.8 (6.7)	<0.001
NYHA class, *n* (%)			<0.001
II	1995 (70.4)	1208 (63.2)	
III	816 (28.8)	682 (35.7)	
IV	23 (0.8)	20 (1.0)	
KCCQ‐TSS, mean (SD)	74.8 (21.5)	71.9 (22.0)	<0.001
Medical history, *n* (%)			
History of AF	N/A	1818 (95.2)	N/A
History of atrial flutter	N/A	226 (11.8)	N/A
History of either AF or atrial flutter	N/A	1885 (98.7)^a^	N/A
Type of AF/atrial flutter^b^			N/A
Paroxysmal	N/A	678 (36.0)	
Persistent	N/A	308 (16.3)	
Permanent	N/A	899 (47.7)	
AF/atrial flutter on ECG at enrolment	N/A	1128 (59.1)	N/A
Hospitalization for HF	1312 (46.3)	939 (49.2)	0.05
Hypertension	2001 (70.6)	1522 (79.7)	<0.001
Type 2 diabetes	1308 (46.2)	831 (43.5)	0.07
Chronic obstructive pulmonary disease	307 (10.8)	278 (14.6)	<0.001
Previous MI	1391 (49.1)	701 (36.7)	<0.001
Previous stroke or TIA	285 (10.1)	297 (15.5)	<0.001
Peripheral artery disease	402 (14.2)	247 (12.9)	0.22
Treatment, *n* (%)			
ACEI/ARB	2400 (84.7)	1552 (81.3)	0.002
ARNI	287 (10.1)	221 (11.6)	0.11
ACEI/ARB/ARNI	2675 (94.4)	1767 (92.5)	0.01
Beta‐blocker	2739 (96.6)	1819 (95.2)	0.01
MRA	2066 (72.9)	1304 (68.3)	<0.001
Ivabradine	202 (7.1)	26 (1.4)	<0.001
Digoxin	326 (11.5)	561 (29.4)	<0.001
Amiodarone	248 (8.8)	321 (16.8)	<0.001
Class I antiarrhythmic drugs	6 (0.2)	9 (0.5)	0.12
Sotalol	17 (0.6)	28 (1.5)	0.003
Oral anticoagulant^c^	372 (13.1)	1597 (83.6)	<0.001
Antiplatelet^d^	2018 (71.2)	574 (30.1)	<0.001
CRT‐P/CRT‐D	178 (6.3)	176 (9.2)	<0.001
ICD/CRT‐D	670 (23.6)	572 (29.9)	<0.001
CHA_2_DS_2_‐VASc score, mean (SD)	4.0 (1.6)	4.3 (1.6)	<0.001
CHA_2_DS_2_‐VASc score ≥ 2, *n* (%)	2690 (94.9)	1850 (96.9)	0.001

ACE, angiotensin‐converting enzyme; AF, atrial fibrillation; ARB, angiotensin receptor blocker; ARNI, angiotensin receptor–neprilysin inhibitor; BMI, body mass index; CRT‐D, cardiac resynchronization therapy‐defibrillator; CHA_2_DS_2_‐VASc, congestive heart failure, hypertension, age ≥ 75 years; diabetes mellitus, prior stroke or transient ischaemic attack or thromboembolism, vascular disease, age 65–74 years, sex category (female); CRT‐P, cardiac resynchronization therapy‐pacemaker; ECG, electrocardiogram; eGFR, estimated glomerular filtration rate; HF, heart failure; ICD, implantable cardioverter‐defibrillator; IQR, interquartile range; KCCQ‐TTS, Kansas City Cardiomyopathy Questionnaire total symptom score; LVEF, left ventricular ejection fraction; MI, myocardial infarction; MRA, mineralocorticoid receptor antagonist; N/A, not appropriate; NT‐proBNP, N‐terminal pro‐B‐type natriuretic peptide; NYHA, New York Heart Association; SD, standard deviation; TIA, transient ischaemic attack.

^a^ The remaining 1.3% had AF on ECG without a history of AF.

^b^ Only recorded for patients with a history of either AF or atrial flutter.

^c^ Vitamin K antagonists (warfarin/coumadin) and direct oral anticoagulants (dabigatran, rivaroxaban, apixaban, and edoxaban).

^d^ Aspirin, adenosine diphosphate receptor inhibitors (clopidogrel, ticagrelor, prasugrel), and adenosine reuptake inhibitors (dipyridamole).

#### Atrial fibrillation on baseline electrocardiogram

A total of 1128 (23.8%) patients had AF on their ECG at enrolment (irrespective of history of AF). The baseline characteristics of these patients compared to patients without any AF are presented in online supplementary *Table* **S1**. The differences were similar to those described above and in *Table* *1*.

#### Type of atrial fibrillation

Baseline characteristics according to the type of AF are presented in online supplementary *Table S2*. Compared to patients with paroxysmal AF, those with persistent/permanent AF had a higher heart rate, glycated haemoglobin concentration, and NT‐proBNP level. Patients with persistent/permanent AF were less likely to have an ischaemic aetiology and had worse NYHA functional class and KCCQ‐TSS than patients with paroxysmal AF. Regarding pharmacological therapy, patients with persistent/permanent AF were more frequently treated with digoxin and an oral anticoagulant, but less often with amiodarone. However, they were substantially less likely to have a defibrillating device, compared to patients with paroxysmal AF.

### Outcomes according to atrial fibrillation status

#### Any atrial fibrillation (history of AF or AF on baseline electrocardiogram)

Patients with any AF had a higher risk of the composite of worsening HF or cardiovascular death. The risk of HF hospitalization was also higher, but the risks of cardiovascular and all‐cause death were not higher, compared with patients without AF (*Table* *2*). However, after adjustment for prognostic variables (both with and without NT‐proBNP) patients with AF had a similar risk for all of these outcomes, compared with those without AF (*Table* *2*).

**Table 2:** Time to first event according to atrial fibrillation (history or baseline electrocardiogram)

	No AF (*n* = 2834)	AF (*n* = 1910)
Worsening HF event or cardiovascular death		
*n* (%)	494 (17.4)	394 (20.6)
Event rate per 100 person‐years (95% CI)	12.7 (11.6–13.8)	14.8 (13.4–16.3)
HR (95% CI)^a^	Reference	1.18 (1.03–1.34)
HR (95% CI)^b^	Reference	1.03 (0.89–1.19)
HR (95% CI)^c^	Reference	0.91 (0.79–1.05)
HF hospitalization or cardiovascular death		
*n* (%)	488 (17.2)	389 (20.4)
Event rate per 100 person‐years (95% CI)	12.5 (11.4–13.7)	14.6 (13.2–16.1)
HR (95% CI)^a^	Reference	1.17 (1.03–1.34)
HR (95% CI)^b^	Reference	1.03 (0.89–1.19)
HR (95% CI)^c^	Reference	0.91 (0.79–1.05)
HF hospitalization		
*n* (%)	287 (10.1)	262 (13.7)
Event rate per 100 person‐years (95% CI)	7.4 (6.5–8.3)	9.8 (8.7–11.1)
HR (95% CI)^a^	Reference	1.34 (1.13–1.59)
HR (95% CI)^b^	Reference	1.10 (0.92–1.32)
HR (95% CI)^c^	Reference	0.97 (0.81–1.17)
Cardiovascular death		
*n* (%)	291 (10.3)	209 (10.9)
Event rate per 100 person‐years (95% CI)	7.1 (6.3–8.0)	7.3 (6.4–8.4)
HR (95% CI)^a^	Reference	1.04 (0.87–1.24)
HR (95% CI)^b^	Reference	0.95 (0.78–1.15)
HR (95% CI)^c^	Reference	0.82 (0.68–1.00)
All‐cause death		
*n* (%)	347 (12.2)	258 (13.5)
Event rate per 100 person‐years (95% CI)	8.5 (7.6–9.4)	9.0 (8.0–10.2)
HR (95% CI)^a^	Reference	1.07 (0.91–1.26)
HR (95% CI)^b^	Reference	0.95 (0.80–1.13)
HR (95% CI)^c^	Reference	0.83 (0.69–0.98)
Stroke		
*n* (%)	54 (1.9)	34 (1.8)
Event rate per 100 person‐years (95% CI)	1.3 (1.0–1.7)	1.2 (0.9–1.7)
HR (95% CI)^a^	Reference	0.91 (0.59–1.40)
HR (95% CI)^b^	Reference	0.84 (0.52–1.34)
HR (95% CI)^c^	Reference	0.80 (0.50–1.28)

AF, atrial fibrillation; CI, confidence interval; HF, heart failure; HR, hazard ratio.

^a^ Cause‐specific Cox regression models stratified according to diabetes mellitus status and adjusted for a history of HF hospitalization and randomized treatment allocation. No adjustment for HF hospitalization in model for all‐cause death.

^b^ Cause‐specific Cox regression models stratified according to diabetes mellitus status and adjusted for a history of HF hospitalization, randomized treatment allocation, age, sex, geographical region, heart rate, systolic blood pressure, body mass index, HF aetiology, left ventricular ejection fraction, New York Heart Association functional class, estimated glomerular filtration rate, hypertension, chronic obstructive pulmonary disease, and prior stroke/transient ischaemic attack. No adjustment for HF hospitalization in model for all‐cause death.

^c^ Fully adjusted cause‐specific Cox regression models, including adjustment for N‐terminal pro‐B‐type natriuretic peptide.

#### Atrial fibrillation on baseline electrocardiogram

After adjustment for prognostic variables (both with and without NT‐proBNP), patients with AF on their baseline ECG had a similar risk of each clinical outcome, compared to individuals without AF (online supplementary *Table S3*).

#### Type of atrial fibrillation

Both patients with paroxysmal AF and patients with persistent/permanent AF had a higher risk of the composite of worsening HF or cardiovascular death than patients without AF. This difference was driven by a higher risk of HF hospitalization, but the risks of cardiovascular and all‐cause death were not higher, compared to patients without AF (online supplementary *Table S4*). However, after adjustment for prognostic variables (both with and without NT‐proBNP), patients with paroxysmal AF and persistent/permanent AF had a similar risk of each clinical outcome, compared to individuals without AF (online supplementary *Table S4*). The unadjusted and adjusted risks in patients with paroxysmal AF were similar to those in patients with persistent/permanent AF.

### Effects of dapagliflozin according to atrial fibrillation status

#### Any atrial fibrillation (history of AF or AF on baseline electrocardiogram)

**Figure 1:**
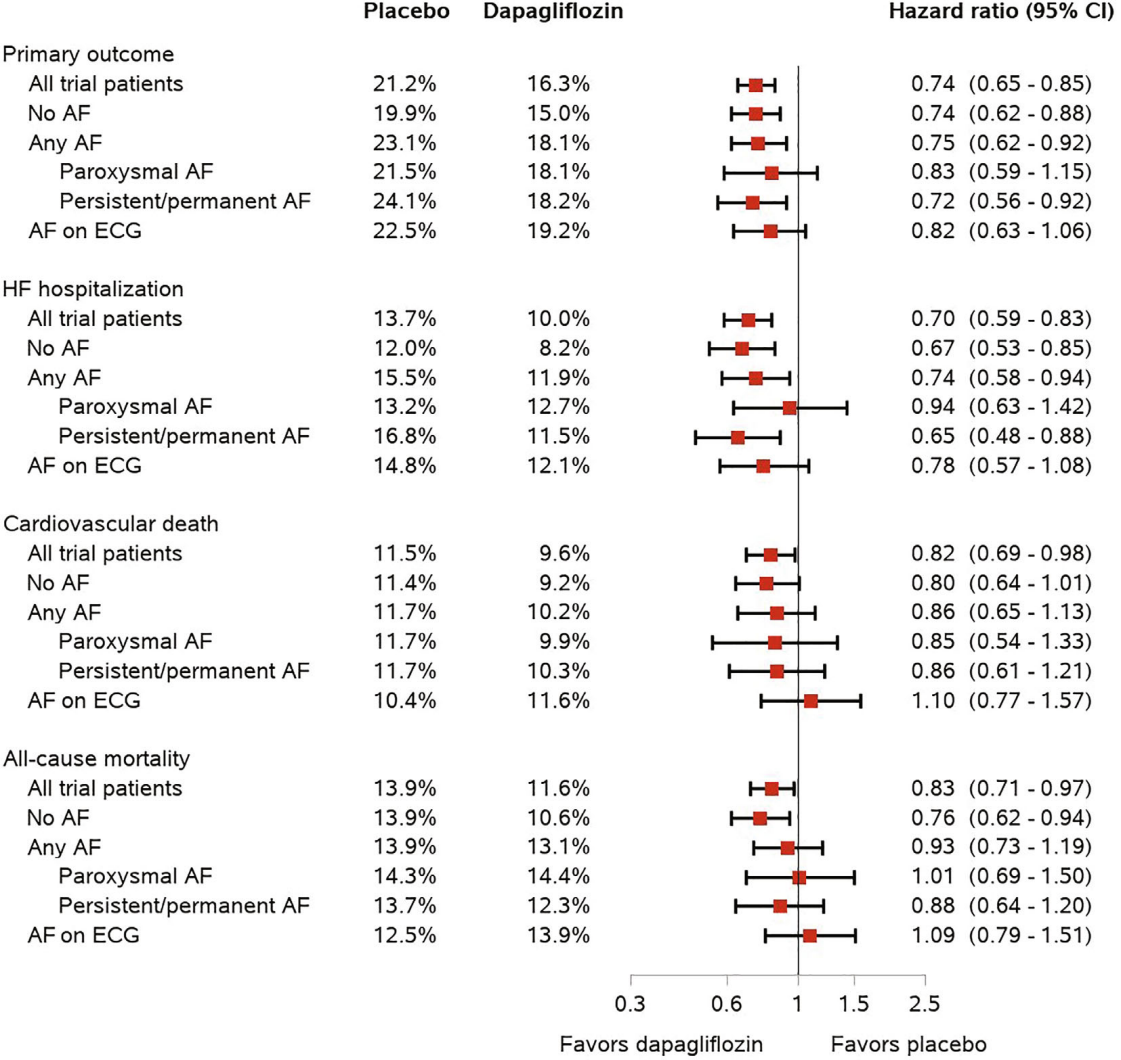
Effects of dapagliflozin compared with placebo on clinical events according to atrial fibrillation (AF) status at baseline. All hazard ratios are adjusted for history of heart failure hospitalization (apart from all‐cause death) and stratified by diabetes status. CI, confidence interval; ECG, electrocardiogram.

Hazard ratios, rate ratios, and odds ratios for the effect of dapagliflozin compared with placebo on the primary and secondary endpoints according to AF status are displayed in *Table* *3*. Dapagliflozin reduced the risk of worsening HF or cardiovascular death to the same extent in patients with (HR 0.75, 95% CI 0.62–0.92) and without AF (HR 0.74, 95% CI 0.62–0.88), with no interaction between AF status and effect of treatment (*p*_interaction_ = 0.88) (*Figure* *1*). The effect of dapagliflozin was consistent in patients with and without AF for all secondary endpoints (*Table* *3*).

**Table 3:** Effects of dapagliflozin compared with placebo on clinical events according to atrial fibrillation (history or baseline electrocardiogram)

Outcome	No AF (*n* = 2834)		AF (*n* = 1910)		*p*‐value for interaction
	Placebo (*n* = 1415)	Dapagliflozin (*n* = 1419)	Placebo (*n* = 956)	Dapagliflozin (*n* = 954)	
Worsening HF event or cardiovascular death					0.88
*n* (%)	281 (19.9)	213 (15.0)	221 (23.1)	173 (18.1)	
Event rate per 100 person‐years (95% CI)	14.7 (13.0–16.5)	10.8 (9.4–12.3)	16.9 (14.8–19.3)	12.7 (11.0–14.8)	
HR (95% CI)	0.74 (0.62–0.88)		0.75 (0.62–0.92)		
HF hospitalization or cardiovascular death					0.97
*n* (%)	276 (19.5)	212 (14.9)	219 (22.9)	170 (17.8)	
Event rate per 100 person‐years (95% CI)	14.3 (12.7–16.1)	10.7 (9.4–12.3)	16.7 (14.6–19.1)	12.5 (10.7–14.5)	
HR (95% CI)	0.75 (0.63–0.89)		0.75 (0.61–0.91)		
HF hospitalization					0.58
*n* (%)	170 (12.0)	117 (8.2)	148 (15.5)	114 (11.9)	
Event rate per 100 person‐years (95% CI)	8.8 (7.6–10.3)	5.9 (4.9–7.1)	11.3 (9.6–13.3)	8.4 (7.0–10.1)	
HR (95% CI)	0.67 (0.53–0.85)		0.74 (0.58–0.94)		
Cardiovascular death					0.70
*n* (%)	161 (11.4)	130 (9.2)	112 (11.7)	97 (10.2)	
Event rate per 100 person‐years (95% CI)	7.9 (6.8–9.2)	6.3 (5.3–7.5)	7.9 (6.6–9.5)	6.8 (5.6–8.3)	
HR (95% CI)	0.80 (0.64–1.01)		0.86 (0.65–1.13)		
All‐cause death					0.22
*n* (%)	196 (13.9)	151 (10.6)	133 (13.9)	125 (13.1)	
Event rate per 100 person‐years (95% CI)	9.6 (8.4–11.1)	7.3 (6.3–8.6)	9.4 (7.9–11.1)	8.7 (7.3–10.4)	
HR (95% CI)	0.76 (0.62–0.94)		0.93 (0.73–1.19)		
Recurrent HF hospitalization or cardiovascular death					0.43
No. of events	408	294	334	273	
RR (95% CI)	0.71 (0.58–0.87)		0.80 (0.64–1.00)		
KCCQ‐TSS					
Change in KCCQ‐TSS score at 8 months	3.3 (2.2–4.4)	5.8 (4.8–6.9)	3.3 (1.9–4.7)	6.5 (5.2–7.8)	0.45
≥5‐point improvement in KCCQ‐TSS at 8 months					0.73
Proportion of patients	52.2	59.1	49.1	57.1	
OR (95% CI)	1.14 (1.06–1.24)		1.17 (1.06–1.29)		
≥5‐point decrease in KCCQ‐TSS at 8 months					0.26
Proportion of patients	31.8	25.5	34.5	25.0	
OR (95% CI)	0.86 (0.79–0.94)		0.80 (0.72–0.88)		

AF, atrial fibrillation; CI, confidence interval; HF, heart failure; HR, hazard ratio; KCCQ‐TSS, Kansas City Cardiomyopathy Questionnaire total symptom score; OR, odds ratio; RR, rate ratio.

The proportions of patients who discontinued trial treatment or experienced adverse events according to treatment assignment were similar, irrespective of AF status (*Table* *4*).

**Table 4:** Adverse events of dapagliflozin compared with placebo according to atrial fibrillation (history or baseline electrocardiogram)

Adverse event	No AF (*n* = 2829)		AF (*n* = 1907)		*p*‐value for interaction
	Placebo (*n* = 1413)	Dapagliflozin (*n* = 1416)	Placebo (*n* = 955)	Dapagliflozin (*n* = 952)	
Discontinuation of study drug for any reason	144 (10.2)	144 (10.2)	114 (11.9)	105 (11.0)	0.65
Discontinuation of study drug due to adverse event	64 (4.5)	60 (4.2)	52 (5.4)	51 (5.4)	0.85
Volume depletion	83 (5.9)	101 (7.1)	79 (8.3)	77 (8.1)	0.31
Renal adverse event	100 (7.1)	77 (5.4)	70 (7.3)	76 (8.0)	0.11
Fracture	27 (1.9)	26 (1.8)	23 (2.4)	23 (2.4)	0.91
Amputation	6 (0.4)	10 (0.7)	6 (0.6)	3 (0.3)	0.17
Major hypoglycaemia	1 (0.1)	2 (0.1)	3 (0.3)	2 (0.2)	0.47
Diabetic ketoacidosis	0 (0.0)	1 (0.1)	0 (0.0)	2 (0.2)	N/A

Values are given as *n* (%). A total of eight randomized patients were excluded from the safety analysis, as these were performed in patients who had undergone randomization and received at least one dose of dapagliflozin or placebo.

AF, atrial fibrillation; N/A, not appropriate.

#### Atrial fibrillation on baseline electrocardiogram

The effect of dapagliflozin in patients with AF on their baseline ECG compared to patients without AF was also consistent for the primary and secondary endpoints (online supplementary *Table S5*). Rates of treatment discontinuation and adverse events in the dapagliflozin and placebo groups were similar in patients with and without AF on their baseline ECG (online supplementary *Table S6*).

#### Type of atrial fibrillation

The effect of dapagliflozin was also consistent for the primary and secondary endpoints according to the type of AF (online supplementary *Table S7*). Similarly, the proportions of patients who discontinued trial treatment or experienced adverse events according to treatment assignment were similar, irrespective of the type of AF (online supplementary *Table S8*).

### New‐onset atrial fibrillation

Among the 2834 patients without AF at baseline, 123 (4.3%) patients developed new‐onset AF during the median follow‐up of 18.2 months. Among patients with incident AF, the arrhythmia was documented on an ECG in 87.7% of participants. Regarding treatment, 30.3% were treated with rate control therapy, 31.1% with an antiarrhythmic drug, 15.6% with electric cardioversion and 4.9% with invasive antiarrhythmic approach; 60.7% received an anticoagulant (online supplementary *Table S9*).

Baseline characteristics according to the development of new‐onset AF are presented in online supplementary *Table S10*. Compared to patients who did not develop new‐onset AF, those who did were older, more often white, more likely to have hypertension, peripheral artery disease, and prior myocardial infarction, and had a higher CHA_2_DS_2_‐VASc score and baseline NT‐proBNP, and lower eGFR. Patients who developed new‐onset AF had a longer duration of HF, but similar LVEF and NYHA functional class compared with those who did not develop new‐onset AF. Regarding background HF therapy, patients who developed new‐onset AF were more likely to have received a defibrillating device.

Among patients who developed new‐onset AF, 37 patients subsequently had a worsening HF event or died from cardiovascular causes, of whom 15 patients experienced this outcome on the exact same day as they are diagnosed with new‐onset AF. New‐onset AF was associated with a higher risk of worsening HF or cardiovascular death (HR 5.44, 95% CI 3.84–7.70), HF hospitalization or cardiovascular death (HR 5.59, 95% CI 3.97–7.88), HF hospitalization (HR 6.24, 95% CI 4.07–9.56), cardiovascular death (HR 4.55, 95% CI 2.92–7.09), and all‐cause death (HR 5.64, 95% CI 3.89–8.17), but not stroke (HR 0.93, 95% CI 0.13–6.85).

**Figure 2:**
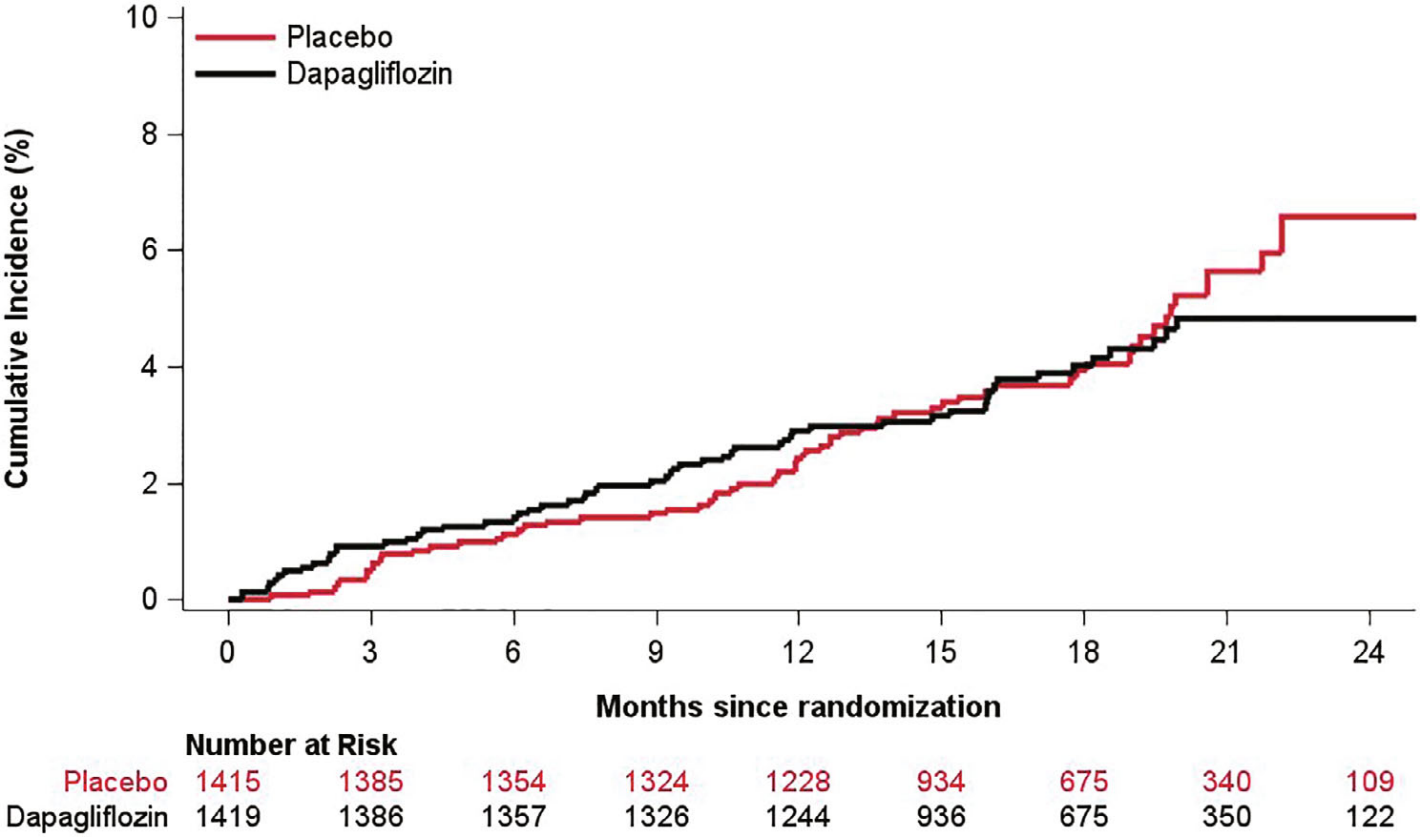
Cumulative incidence of new‐onset atrial fibrillation in dapagliflozin according to randomized treatment assignment in patients without atrial fibrillation (history or baseline electrocardiogram). Cumulative incidence of new‐onset atrial fibrillation was estimated using the Aalen–Johansen estimator, taking the competing risk of death into account.

Overall, 66 (4.7%) and 57 (4.0%) patients in the placebo and dapagliflozin group, respectively, developed new‐onset AF, corresponding to a rate of 3.3 and 2.8 events per 100 person‐years, respectively (*Figure* *2*). Compared with placebo, dapagliflozin did not significantly reduce the risk of new‐onset AF (HR 0.86, 95% CI 0.60–1.22). Fine–Gray competing risk analysis, accounting for the competing risk of death, yielded a similar finding (subdistribution HR 0.87, 95% CI 0.61–1.24).

## Discussion

In DAPA‐HF, AF at enrolment (irrespective of definition or type) was not associated with a higher risk of adverse outcomes. In addition, dapagliflozin, compared with placebo, reduced the risk of worsening HF events, cardiovascular death, and all‐cause death, and improved symptoms, in patients with and without AF (irrespective of definition or type). However, dapagliflozin did not significantly reduce the risk of new‐onset AF (*Graphical Abstract*).

### Baseline characteristics and outcomes according to atrial fibrillation

In DAPA‐HF, there were substantial differences in the clinical profile between HFrEF patients with and without any AF (and between type of AF), most of which confirmed differences reported in prior HFrEF trials.^3^, ^4^ One of these remains a concern. There was persisting underuse of oral anticoagulants (74%) in patients with paroxysmal AF despite >95% having a CHA_2_DS_2_‐VASc score ≥ 2 (by contrast 90% of patients with persistent/permanent AF received an anticoagulant).^29^

The differences in the clinical profile between HFrEF patients with and without AF have fuelled a vigorous debate about whether AF is an independent predictor of adverse outcomes or simply a marker of more advanced disease in sicker patients. The conflicting findings in previous studies may reflect residual confounding, particularly from lack of adjustment for NT‐proBNP, the single strongest predictor of outcomes in HFrEF.^1^, ^3^, ^4^, ^5^, ^6^, ^7^, ^8^, ^9^, ^10^, ^11^, ^12^ We found that AF (irrespective of definition) at baseline was no longer associated with a higher risk of any of the outcomes of interest after adjustment for known prognostic variables. This was the case whether NT‐proBNP was or was not included in the adjustment. Our findings are in line with recent data from the Vericiguat Global Study in Subjects With Heart Failure With Reduced Ejection Fraction trial (VICTORIA).^4^ These results from two of the most contemporary trials in HFrEF do not support the view that AF is an independent prognostic factor. However, based on our analysis, the view that AF is a marker of more advanced disease seems only partially true. The excess risk observed in patients with AF was related to hospital admission and not mortality. Despite having higher NT‐proBNP levels, patients with AF did not have higher all‐cause mortality, even in the unadjusted analyses. Of course, these new findings have been made in patients receiving excellent background pharmacological therapy and this may be an important distinction from older studies.

### Efficacy and safety of dapagliflozin according to baseline atrial fibrillation

While AF does not appear to modify the effects of angiotensin receptor blockers, sacubitril/valsartan, mineralocorticoid receptor antagonists, or vericiguat in patients with HFrEF,^4^, ^5^, ^6^ patients with AF obtain less benefit from beta‐blockers compared to those without AF.^15^ Recently, a diminished effect of omecamtiv mecarbil has also been reported in patients with AF, compared to sinus rhythm.^16^ In light of these differences, it is important to examine the efficacy of new treatments in HFrEF patients with and without AF. We found the efficacy of dapagliflozin was not modified by AF, irrespective of the definition or type of AF. Specifically, dapagliflozin, compared with placebo, reduced the risk of worsening HF or cardiovascular death to a similar extent in patients with and without AF. The benefits of dapagliflozin on HF hospitalization (both first and recurrent), cardiovascular death, and all‐cause death were also consistent, irrespective of AF status.

A further fundamental goal of the management of HFrEF is to reduce symptoms and improve quality of life and this is even more important in patients with AF who have a greater symptom burden than those without, as confirmed by the KCCQ‐TSS findings in the present study. More patients treated with dapagliflozin had a clinically meaningful improvement, and fewer a deterioration, in symptoms (≥5 point change in KCCQ‐TSS), and this benefit over placebo was similar in patients with and without AF at baseline (again irrespective of definition and type). In addition, dapagliflozin was as well‐tolerated and safe in patients with AF as in those without. Dapagliflozin is, therefore, a useful additional therapy for HFrEF patients with AF.

### New‐onset atrial fibrillation

The importance of new‐onset AF is much less well studied in patients with HFrEF. Two recent analyses suggested that new‐onset AF confers a high risk of adverse outcomes, including HF hospitalization and death, and we confirmed this in the present study, with a striking 5 to 6‐fold higher risk of non‐fatal and fatal outcomes when compared to patients without incident AF.^3^, ^4^, ^5^, ^11^, ^19^, ^20^ Our findings in the present trial [and in the Aliskiren Trial to Minimize Outcomes in Patients with Heart Failure (ATMOSPHERE)^30^ and the Prospective Comparison of Angiotensin Receptor–Neprilysin Inhibitor with Angiotensin‐Converting Enzyme Inhibitor to Determine Impact on Global Mortality and Morbidity in Heart Failure trial (PARADIGM‐HF)^31^] contrast with those recently described in the VICTORIA trial which enrolled sicker patients who had recently been hospitalized. In VICTORIA, the incidence of AF was much higher, but the risk associated with new‐onset AF was not as great.^4^ The finding that new‐onset AF is associated with worse outcomes in the present study, whereas a history of AF (or AF at baseline) is not, may seem counterintuitive. However, it is likely that some episodes of new‐onset AF with a rapid ventricular rate may lead to haemodynamic decompensation and subsequent hospital admission.^4^ Based on the present and previous similar findings, it appears to be important to try and prevent the development of new‐onset AF. Renin–angiotensin system inhibitors, beta‐blockers, and mineralocorticoid receptor antagonists have each been shown to reduce incident AF, and the low rate of new‐onset AF in the present trial may reflect the high usage of these drugs in DAPA‐HF.^6^, ^15^, ^21^, ^22^, ^23^ By contrast, neprilysin inhibition and vericiguat were recently shown not to reduce incident AF in patients with HFrEF.^4^, ^31^ While this may also be true for SGLT2 inhibitors, based on the present findings, it is also possible that a difference from placebo was not detected because of the low statistical power due to the small number of episodes of incident AF and relatively short duration of follow‐up in DAPA‐HF. We raise this possibility because a *post hoc* analysis of the DECLARE‐TIMI 58 trial, which had nearly five times more AF events and follow‐up through 4 years, did show that dapagliflozin reduced the number of episodes of AF, with the curves particularly diverging after 2 years, albeit in a quite different patient population.^24^ Indeed, the point estimate we observed in DAPA‐HF (HR 0.86) is consistent with that from the DECLARE‐TIMI58 trial (HR 0.81, 95% CI 0.68–0.95). Moreover, three recent cardiac remodelling trials, collectively, showed that SGLT2 inhibition reduced left atrial size in patients with HFrEF, an effect supporting the possibility of a beneficial effect on incident AF.^32^, ^33^, ^34^ However, given the exploratory nature of the present analysis (and the *post hoc* analysis from the DECLARE‐TIMI 58 trial) as well as the lack of adjudication and systematic screening for new‐onset AF in both trials, the potential anti‐fibrillatory effect of SGLT2 inhibitors merits further investigation in a prospective manner, perhaps using systematic ECG monitoring which is likely to detect a higher incidence of new‐onset AF. With several studies now highlighting the very high risk associated with incident AF, new strategies to detect and treat this problem early may also be worthwhile.^35^

### Limitations

This study has some limitations. Systematic ECG monitoring would likely have detected AF in some patients with no investigator‐reported history (and of new‐onset AF). Similarly, among those with known AF, some degree of misclassification of the type of AF is likely. Information on the duration of AF at enrolment was not available. New‐onset AF was said not to have been documented on an ECG in 15 of the 123 cases (13 of these 15 had paroxysmal AF). Presumably these cases were identified by clinical examination, on an ECG monitor rhythm strip or by an implanted device. The duration of follow‐up in DAPA‐HF was relatively short, and this may, in part, explain the low number of patients with new‐onset AF and stroke. Finally, although it would have been interesting to examine the long‐term effects of dapagliflozin on cardiac remodelling, specifically left atrial size, echocardiographic data were not available in the present study although have been reported in other trials.^32^, ^33^, ^34^

## Conclusions

In DAPA‐HF, dapagliflozin, compared with placebo, reduced the risk of worsening HF events, cardiovascular death, and all‐cause death, and improved symptoms, in patients with and without AF (irrespective of definition or type). Dapagliflozin did not significantly reduce the risk of new‐onset AF.

## Funding

The DAPA‐HF trial was funded by AstraZeneca. Prof. McMurray is supported by British Heart Foundation Centre of Research Excellence Grant RE/18/6/34217.

**Conflict of interest**: J.H.B. reports advisory board honoraria from Bayer, outside the submitted work. K.F.D. reports compensation from AstraZeneca for other services. P.S.J. reports compensation for consultant services from Novo Nordisk AS, Novartis, AstraZeneca and Boehringer Ingelheim. R.A.d.B. reports grants from Cardior Pharmaceuticals GmbH to other; compensation for other services from Novartis, AstraZeneca, Roche Diagnostics GmbH, Bayer, Abbott Fund; grants from AstraZeneca, Ionis Pharmaceuticals, Inc., Roche Diagnostics GmbH, Boehringer Ingelheim, Novo Nordisk and Abbott Fund to other. M.B. received personal fees from Abbott, Amgen, AstraZeneca, Bayer, Boehringer Ingelheim, Servier, Medtronic, Vifor, and Novartis. A.S.D. reports receiving personal fees from Abbott, Biofourmis, Boston Scientific, Boehringer Ingelheim, Merck, Regeneron, and Relypsa and grants and personal fees from AstraZeneca, Alnylam, and Novartis. J.G.H. reports receiving grants and personal fees from AstraZeneca Canada and Boerhinger Ingelheim/Eli Lilly during the conduct of the study and grants and personal fees from Servier Canada, Novartis, Pfizer, and Bayer; personal fees from Otsuka, Alnylam, and Akcea; grants from Medtronic; and serving on the medical advisory board for Caridiol outside the submitted work. S.E.I. reports compensation for consultant services from Merck, Lexicon Pharmaceuticals, Inc., AstraZeneca, Boehringer Ingelheim, Abbott Diabetes Care, VTV Therapeutics, Novo Nordisk, Esperion; travel support from Novo Nordisk, Boehringer Ingelheim, AstraZeneca; compensation for other services from AstraZeneca, Boehringer Ingelheim. M.N.K. reports compensation for consultant services from Novo Nordisk, Applied Therapeutics, Janssen Global Services, LLC, AstraZeneca, Eli Lilly and Company, Merck, Bayer, Amgen, Sanofi US Services Inc, Boehringer Ingelheim, Vifor Pharma; grants from Boehringer Ingelheim, AstraZeneca. F.A.M. reports personal fees from AstraZeneca. J.C.N. reports receiving grants from AstraZeneca during the conduct of the study and personal fees from Amgen, Daiichi‐Sankyo, and Servier, grants from AstraZeneca, Bristol‐Myers Squibb, CLS Behring, Dalcor, Jansen, Novo Nordisk, and Vifor, and grants and personal fees from Bayer, Novartis, and Sanofi. M.C.P. reports compensation for endpoint review committee services from Bayer, Boehringer Ingelheim, Takeda California, Inc, Novo Nordisk; compensation for consultant services from Boehringer Ingelheim, AstraZeneca, Novo Nordisk, Novartis, SQ Innovation; grants from Boehringer Ingelheim, Novartis, SQ Innovation and AstraZeneca to other. P.P. reports compensation for consultant services from AstraZeneca, Boehringer Ingelheim, Servier, Amgen, Vifor Pharma; compensation for other services from AstraZeneca, Pfizer, Novartis, Abbott Vascular. O.B. reports employment by AstraZeneca AB. A.M.L. reports stock holdings in AstraZeneca and employment by AstraZeneca. M. Sjöstrand reports employment by AstraZeneca. S.D.S. reports compensation for consultant services from GlaxoSmithKline, Arena, Novartis, Quantum Genomics, Bayer, Bristol‐Myers Squibb, Cardior, Cytokinetics, Sarepta Therapeutics, Action Medical Research, Tremeau, Theracos, Moderna, Akros, Amgen, Boehringer Ingelheim, Alnylam Pharmaceuticals, Roche Diagnostics Corporation, American Regent, AstraZeneca, Cardurion, Merck, Daiichi Sankyo, Myokardia, Sanofi Pasteur Inc, Abbott Vascular, Eli Lilly and Company, Johnson and Johnson, CellProThera, Tenaya, Cardiac Dimensions; grants from Theracos, Celladon Corporation, Bristol‐Myers Squibb, GlaxoSmithKline, Amgen, Respicardia, Inc, Novo Nordisk, Bayer, AstraZeneca, Mesoblast, Myokardia, Actelion Pharmaceuticals, Alnylam Pharmaceuticals, Bellepheron, Ionis, Sanofi Pasteur Inc, Neurotronik, Cytokinetics, Eli Lilly and Company, Novartis, Eidos; stock options in Dinaqor; travel support from Corvia. M.S.S. reports research grant support through Brigham and Women's Hospital from: Abbott, Amgen, Anthos Therapeutics, AstraZeneca, Bayer, Daiichi‐Sankyo, Eisai, Intarcia, IONIS, Medicines Company, MedImmune, Merck, Novartis, Pfizer, Quark Pharmaceuticals; and consulting for Althera, Amgen, Anthos Therapeutics, AstraZeneca, Bristol‐Myers Squibb, CVS Caremark, DalCor, Dr. Reddy's Laboratories, Fibrogen, IFM Therapeutics, Intarcia, MedImmune, Merck, Moderna, Novo Nordisk. J.J.V.McM. reports grants from AstraZeneca. L.K. reports compensation for other services from Novartis, Novo Nordisk, AstraZeneca.

## Supporting information


**Table S1.** Baseline characteristics of the study population: no AF (history or baseline ECG) vs. AF on ECG (irrespective of history).
**Table S2.** Baseline characteristics of the study population according to type of AF.
**Table S3.** Time to first event: no AF (history or baseline ECG) vs. AF on ECG (irrespective of history).
**Table S4.** Time to first event according to type of AF.
**Table S5.** Effects of dapagliflozin compared with placebo on clinical events: no AF (history or baseline ECG) vs. AF on ECG (irrespective of history).
**Table S6.** Adverse events of dapagliflozin compared with placebo: no AF (history or baseline ECG) versus AF on ECG (irrespective of history)
**Table S7.** Effects of dapagliflozin compared with placebo on clinical events according to type of AF.
**Table S8.** Adverse events of dapagliflozin compared with placebo according to type of AF.
**Table S9.** Characteristics of new‐onset AF status in patients without AF (history or baseline ECG).
**Table S10.** Baseline characteristics by new‐onset AF status in patients without AF (history or baseline ECG).
