# Supplementary material for: Dapagliflozin and atrial fibrillation in heart failure with reduced ejection fraction: insights from DAPA‐HF
Source: Eur J Heart Fail. 2021 Nov 24;24(3):513–25. doi: 10.1002/ejhf.2381 (PMC10234442; doi:10.1002/ejhf.2381)
Supplement: Supplementary file 1 — Table S1. Baseline characteristics of the study population: no AF (history or baseline ECG) vs. AF on ECG (irrespective of history). Table S2. Baseline characteristics of the study population according to type of AF. Table S3. Time to first event: no AF (history or baseline ECG) vs. AF on ECG (irrespective of history). Table S4. Time to first event according to type of AF. Table S5. Effects of dapagliflozin compared with placebo on clinical events: no AF (history or baseline ECG) vs. AF on ECG (irrespective of history). Table S6. Adverse events of dapagliflozin compared with placebo: no AF (history or baseline ECG) versus AF on ECG (irrespective of history) Table S7. Effects of dapagliflozin compared with placebo on clinical events according to type of AF. Table S8. Adverse events of dapagliflozin compared with placebo according to type of AF. Table S9. Characteristics of new‐onset AF status in patients without AF (history or baseline ECG). Table S10. Baseline characteristics by new‐onset AF status in patients without AF (history or baseline ECG). [file EJHF-24-513-s001.docx]

**eTable 1. Baseline characteristics of the study population: No AF (history or baseline ECG) versus AF on ECG (irrespective of history)**

|  | No AF  N=2,834 | AF on ECG  N=1,128 | P-value |
| --- | --- | --- | --- |
| Age (years), mean (SD) | 64.3 (11.2) | 69.5 (9.5) | <0.001 |
| Sex, N (%) |  |  | 0.008 |
| Female | 713 (25.2) | 239 (21.2) |  |
| Male | 2,121 (74.8) | 889 (78.8) |  |
| Race, N (%) |  |  | <0.001 |
| Asian | 806 (28.4) | 202 (17.9) |  |
| Black | 156 (5.5) | 34 (3.0) |  |
| White | 1,825 (64.4) | 876 (77.7) |  |
| Other | 47 (1.7) | 16 (1.4) |  |
| Geographic region, N (%) |  |  | <0.001 |
| Asia/Pacific | 791 (27.9) | 200 (17.7) |  |
| Europe | 1,085 (38.3) | 640 (56.7) |  |
| North America | 391 (13.8) | 106 (9.4) |  |
| South America | 567 (20.0) | 182 (16.1) |  |
| Physiologic measures |  |  |  |
| Systolic blood pressure (mmHg), mean (SD) | 121.9 (16.8) | 122.2 (15.0) | 0.66 |
| Heart rate (bpm), mean (SD) | 70.7 (10.8) | 75.7 (13.3) | <0.001 |
| BMI (kg/m^2^), mean (SD) | 27.7 (5.9) | 28.9 (6.1) | <0.001 |
| Creatinine (μmol/L), mean (SD) | 101.7 (30.6) | 106.9 (28.8) | <0.001 |
| Glycated haemoglobin, mean (SD) | 6.1 (5.7-7.0) | 6.1 (5.7-6.7) | 0.73 |
| eGFR (mL/min/1.73m^2^), mean (SD) | 68.5 (20.1) | 62.4 (17.4) | <0.001 |
| eGFR (mL/min/1.73m^2^), N (%) |  |  | <0.001 |
| < 60 | 1,001 (35.3) | 522 (46.3) |  |
| > 60 | 1,831 (64.7) | 606 (53.7) |  |
| NT-proBNP, median (IQR) | 1,242 (742-2,325) | 1,948 (1,265-3,204) | <0.001 |
| Main cause of HF, N (%) |  |  | <0.001 |
| Ischemic | 1,723 (60.8) | 510 (45.2) |  |
| Non-ischemic | 890 (31.4) | 508 (45.0) |  |
| Unknown | 221 (7.8) | 110 (9.8) |  |
| Duration of HF, N (%) |  |  | 0.002 |
| 0-3 months | 94 (3.3) | 48 (4.3) |  |
| 3-6 months | 260 (9.2) | 88 (7.8) |  |
| 6-12 months | 376 (13.3) | 117 (10.4) |  |
| 1-2 years | 450 (15.9) | 151 (13.4) |  |
| 2-5 years | 652 (23.0) | 263 (23.3) |  |
| >5 years | 1,002 (35.4) | 461 (40.9) |  |
| LVEF, mean (SD) | 30.5 (6.8) | 32.4 (6.5) | <0.001 |
| NYHA class, N (%) |  |  | <0.001 |
| II | 1,995 (70.4) | 690 (61.2) |  |
| III | 816 (28.8) | 425 (37.7) |  |
| IV | 23 (0.8) | 13 (1.2) |  |
| KCCQ-TSS, mean (SD) | 74.8 (21.5) | 70.8 (22.3) | <0.001 |
| Medical history, N (%) |  |  |  |
| History of atrial fibrillation | N/A | 1,082 (95.9) | N/A |
| History of atrial flutter | N/A | 101 (9.0) | N/A |
| History of either atrial fibrillation or flutter | N/A | 1,103 (97.8)^#^ | N/A |
| Type of atrial fibrillation/flutter* |  |  | N/A |
| Paroxysmal | N/A | 148 (13.4) |  |
| Persistent | N/A | 195 (17.7) |  |
| Permanent | N/A | 760 (68.9) |  |
| Hospitalization for HF | 1,312 (46.3) | 570 (50.5) | 0.02 |
| Hypertension | 2,001 (70.6) | 920 (81.6) | <0.001 |
| Type 2 diabetes | 1,308 (46.2) | 503 (44.6) | 0.37 |
| Chronic obstructive pulmonary disease | 307 (10.8) | 161 (14.3) | 0.003 |
| Previous MI | 1,391 (49.1) | 339 (30.1) | <0.001 |
| Previous stroke or TIA | 285 (10.1) | 169 (15.0) | <0.001 |
| Peripheral artery disease | 402 (14.2) | 129 (11.4) | 0.02 |
| Treatment, N (%) |  |  |  |
| ACEI/ARB | 2,400 (84.7) | 962 (85.3) | 0.64 |
| ARNI | 287 (10.1) | 98 (8.7) | 0.17 |
| ACEI/ARB/ARNI | 2,675 (94.4) | 1,058 (93.8) | 0.47 |
| Beta-blocker | 2,739 (96.6) | 1,073 (95.1) | 0.02 |
| MRA | 2,066 (72.9) | 786 (69.7) | 0.04 |
| Ivabradine | 202 (7.1) | 4 (0.4) | <0.001 |
| Digoxin | 326 (11.5) | 401 (35.5) | <0.001 |
| Amiodarone | 215 (7.6) | 81 (7.2) | 0.66 |
| Class I antiarrythmic drugs | 6 (0.2) | 3 (0.3) | 0.72 |
| Sotalol | 17 (0.6) | 4 (0.4) | 0.34 |
| Oral anticoagulant** | 372 (13.1) | 1,003 (88.9) | <0.001 |
| Antiplatelet*** | 2,018 (71.2) | 277 (24.6) | <0.001 |
| CRT-P/CRT-D | 178 (6.3) | 41 (3.6) | 0.001 |
| ICD/CRT-D | 670 (23.6) | 187 (16.7) | <0.001 |
| CHA_2_DS_2_-VASc score, mean (SD) | 3.9 (1.6) | 4.2 (1.5) | <0.001 |
| CHA_2_DS_2_-VASc score >2, N (%) | 2,680 (94.6) | 1,091 (96.7) | 0.004 |

*ACE angiotensin-converting enzyme; ARB, angiotensin receptor blocker; ARNI, angiotensin receptor neprilysin inhibitor; BMI, body mass index; CRT-D, cardiac resynchronization therapy–defibrillator; CHA_2_DS_2_-VASc, congestive heart failure, hypertension, age >75 years; diabetes mellitus, prior stroke or transient ischemic attack or thromboembolism, vascular disease, age 65–74 years, sex category; CRT-P, cardiac resynchronization therapy–pacemaker; eGFR, estimated glomerular filtration rate; HF, heart failure; ICD, implantable cardioverter-defibrillator; IQR, interquartile range; KCCQ-TTS, Kansas City Cardiomyopathy Questionnaire Total Symptom Score; MI, myocardial infarction; NT-proBNP, N-terminal pro-B-type natriuretic peptide; NYHA, New York Heart Association; TIA, transient ischemic attack.*

**Only recorded for patients with a history of either atrial fibrillation or flutter.*

***Vitamin K antagonists (warfarin/coumadin) and “direct” oral anticoagulants (dabigatran, rivaroxaban, apixaban, and edoxaban).*

****Aspirin, ADP-receptor inhibitors (clopidogrel, ticagrelor, prasugrel), and adenosine reuptake inhibitors (dipyridamole).*

*^#^The remaining 2.2% had AF on ECG without a history of AF.*

**eTable 2. Baseline characteristics of the study population according to type of AF**

|  | No AF (I)  N=2,834 | Paroxysmal  AF (II)  N=703 | Persistent/permanent  AF (III)  N=1,207 | P-value | P-value  (II versus III) |
| --- | --- | --- | --- | --- | --- |
| Age (years), mean (SD) | 64.3 (11.2) | 69.0 (10.0) | 69.5 (9.4) | <0.001 | 0.30 |
| Sex, N (%) |  |  |  | 0.002 | 0.84 |
| Female | 713 (25.2) | 144 (20.5) | 252 (20.9) |  |  |
| Male | 2,121 (74.8) | 559 (79.5) | 955 (79.1) |  |  |
| Race, N (%) |  |  |  | <0.001 | 0.04 |
| Asian | 806 (28.4) | 103 (14.7) | 207 (17.1) |  |  |
| Black | 156 (5.5) | 36 (5.1) | 34 (2.8) |  |  |
| White | 1,825 (64.4) | 557 (79.2) | 951 (78.8) |  |  |
| Other | 47 (1.7) | 7 (1.0) | 15 (1.2) |  |  |
| Geographic region, N (%) |  |  |  | <0.001 | <0.001 |
| Asia/Pacific | 791 (27.9) | 100 (14.2) | 205 (17.0) |  |  |
| Europe | 1,085 (38.3) | 381 (54.2) | 688 (57.0) |  |  |
| North America | 391 (13.8) | 165 (23.5) | 121 (10.0) |  |  |
| South America | 567 (20.0) | 57 (8.1) | 193 (16.0) |  |  |
| Physiologic measures |  |  |  |  |  |
| Systolic blood pressure (mmHg), mean (SD) | 121.9 (16.8) | 121.8 (16.3) | 121.6 (15.3) | 0.84 | 0.81 |
| Heart rate (bpm), mean (SD) | 70.7 (10.8) | 69.1 (12.3) | 74.8 (12.8) | <0.001 | <0.001 |
| BMI (kg/m^2^), mean (SD) | 27.7 (5.9) | 28.6 (5.9) | 28.9 (6.0) | <0.001 | 0.27 |
| Creatinine (μmol/L), mean (SD) | 101.7 (30.6) | 110.0 (31.3) | 107.7 (28.7) | <0.001 | 0.10 |
| Glycated haemoglobin, mean (SD) | 6.1 (5.7-7.0) | 6.0 (5.6-6.7) | 6.1 (5.7-6.8) | 0.002 | 0.002 |
| eGFR (mL/min/1.73m^2^), mean (SD) | 68.5 (20.1) | 61.4 (17.9) | 61.9 (17.4) | <0.001 | 0.53 |
| eGFR (mL/min/1.73m^2^), N (%) |  |  |  | <0.001 | 0.47 |
| < 60 | 1,001 (35.3) | 348 (49.5) | 577 (47.8) |  |  |
| > 60 | 1,831 (64.7) | 355 (50.5) | 630 (52.2) |  |  |
| NT-proBNP, median (IQR) | 1,242 (742-2,325) | 1,485 (867-2,811) | 1,921 (1,237-3,147) | <0.001 | <0.001 |
| Main cause of HF, N (%) |  |  |  | <0.001 | <0.001 |
| Ischemic | 1,723 (60.8) | 414 (58.9) | 537 (44.5) |  |  |
| Non-ischemic | 890 (31.4) | 240 (34.1) | 557 (46.1) |  |  |
| Unknown | 221 (7.8) | 49 (7.0) | 113 (9.4) |  |  |
| Duration of HF, N (%) |  |  |  | <0.001 | 0.30 |
| 0-3 months | 94 (3.3) | 20 (2.8) | 36 (3.0) |  |  |
| 3-6 months | 260 (9.2) | 48 (6.8) | 85 (7.0) |  |  |
| 6-12 months | 376 (13.3) | 53 (7.5) | 126 (10.4) |  |  |
| 1-2 years | 450 (15.9) | 87 (12.4) | 149 (12.4) |  |  |
| 2-5 years | 652 (23.0) | 162 (23.0) | 291 (24.1) |  |  |
| >5 years | 1,002 (35.4) | 333 (47.4) | 520 (43.1) |  |  |
| LVEF, mean (SD) | 30.5 (6.8) | 31.5 (7.0) | 32.1 (6.6) | <0.001 | 0.07 |
| NYHA class, N (%) |  |  |  | <0.001 | 0.01 |
| II | 1,995 (70.4) | 473 (67.3) | 735 (60.9) |  |  |
| III | 816 (28.8) | 221 (31.4) | 461 (38.2) |  |  |
| IV | 23 (0.8) | 9 (1.3) | 11 (0.9) |  |  |
| KCCQ-TSS, mean (SD) | 74.8 (21.5) | 73.6 (21.9) | 70.9 (22.1) | <0.001 | 0.01 |
| Medical history, N (%) |  |  |  |  |  |
| History of atrial fibrillation | N/A | 637 (90.6) | 1,181 (97.8) | N/A | <0.001 |
| History of atrial flutter | N/A | 116 (16.5) | 110 (9.1) | N/A | <0.001 |
| History of either atrial fibrillation or flutter | N/A | 678 (96.4)^#^ | 1,207 (100.0) | N/A | N/A |
| Type of atrial fibrillation/flutter* |  |  |  | N/A | N/A |
| Paroxysmal | N/A | 678 (100.0) | N/A |  |  |
| Persistent | N/A | N/A | 899 (74.5) |  |  |
| Permanent | N/A | N/A | 308 (25.5) |  |  |
| Atrial fibrillation/flutter on ECG at enrolment | N/A | 173 (24.6) | 955 (79.1) | N/A | <0.001 |
| Hospitalization for HF | 1,312 (46.3) | 326 (46.4) | 613 (50.8) | 0.03 | 0.06 |
| Hypertension | 2,001 (70.6) | 551 (78.4) | 971 (80.4) | <0.001 | 0.29 |
| Type 2 diabetes | 1,308 (46.2) | 296 (42.1) | 535 (44.3) | 0.13 | 0.35 |
| Chronic obstructive pulmonary disease | 307 (10.8) | 101 (14.4) | 177 (14.7) | <0.001 | 0.86 |
| Previous MI | 1,391 (49.1) | 327 (46.5) | 374 (31.0) | <0.001 | <0.001 |
| Previous stroke or TIA | 285 (10.1) | 118 (16.8) | 179 (14.8) | <0.001 | 0.25 |
| Peripheral artery disease | 402 (14.2) | 100 (14.2) | 147 (12.2) | 0.21 | 0.20 |
| Treatment, N (%) |  |  |  |  |  |
| ACEI/ARB | 2,400 (84.7) | 548 (78.0) | 1,004 (83.2) | <0.001 | 0.005 |
| ARNI | 287 (10.1) | 103 (14.7) | 118 (9.8) | 0.001 | 0.001 |
| ACEI/ARB/ARNI | 2,675 (94.4) | 646 (91.9) | 1,121 (92.9) | 0.02 | 0.43 |
| Beta-blocker | 2,739 (96.6) | 673 (95.7) | 1,146 (94.9) | 0.03 | 0.44 |
| MRA | 2,066 (72.9) | 467 (66.4) | 837 (69.3) | 0.001 | 0.19 |
| Ivabradine | 202 (7.1) | 20 (2.8) | 6 (0.5) | <0.001 | <0.001 |
| Digoxin | 326 (11.5) | 133 (18.9) | 428 (35.5) | <0.001 | <0.001 |
| Amiodarone | 215 (7.6) | 171 (24.3) | 115 (9.5) | <0.001 | <0.001 |
| Class I antiarrythmic drugs | 6 (0.2) | 5 (0.7) | 4 (0.3) | 0.10 | 0.30 |
| Sotalol | 17 (0.6) | 23 (3.3) | 5 (0.4) | <0.001 | <0.001 |
| Oral anticoagulant** | 372 (13.1) | 519 (73.8) | 1,078 (89.3) | <0.001 | <0.001 |
| Antiplatelet*** | 2,018 (71.2) | 303 (43.1) | 271 (22.5) | <0.001 | <0.001 |
| CRT-P/CRT-D | 178 (6.3) | 87 (12.4) | 89 (7.4) | <0.001 | <0.001 |
| ICD/CRT-D | 670 (23.6) | 297 (42.2) | 275 (22.8) | <0.001 | <0.001 |
| CHA_2_DS_2_-VASc score, mean (SD) | 3.9 (1.6) | 4.3 (1.5) | 4.2 (1.5) | <0.001 | 0.04 |
| CHA_2_DS_2_-VASc score >2, N (%) | 2,680 (94.6) | 682 (97.0) | 1,164 (96.4) | 0.003 | 0.50 |

*ACE angiotensin-converting enzyme; ARB, angiotensin receptor blocker; ARNI, angiotensin receptor neprilysin inhibitor; BMI, body mass index; CRT-D, cardiac resynchronization therapy–defibrillator; CHA_2_DS_2_-VASc, congestive heart failure, hypertension, age >75 years; diabetes mellitus, prior stroke or transient ischemic attack or thromboembolism, vascular disease, age 65–74 years, sex category; CRT-P, cardiac resynchronization therapy–pacemaker; eGFR, estimated glomerular filtration rate; HF, heart failure; ICD, implantable cardioverter-defibrillator; IQR, interquartile range; KCCQ-TTS, Kansas City Cardiomyopathy Questionnaire Total Symptom Score; MI, myocardial infarction; NT-proBNP, N-terminal pro-B-type natriuretic peptide; NYHA, New York Heart Association; TIA, transient ischemic attack.*

**Only recorded for patients with a history of either atrial fibrillation or flutter.*

***Vitamin K antagonists (warfarin/coumadin) and “direct” oral anticoagulants (dabigatran, rivaroxaban, apixaban, and edoxaban).*

****Aspirin, ADP-receptor inhibitors (clopidogrel, ticagrelor, prasugrel), and adenosine reuptake inhibitors (dipyridamole).*

*^#^The remaining 3.6% had AF on ECG without a history of AF.*

**eTable 3. Time to first event: No AF (history or baseline ECG) versus AF on ECG (irrespective of history)**

|  | No AF  N=2,834 | AF on ECG  N=1,128 |
| --- | --- | --- |
| Worsening HF event or cardiovascular death | | |
| N (%) | 494 (17.4) | 235 (20.8) |
| Event rate per 100 person-years (95% CI) | 12.7 (11.6-13.8) | 14.7 (13.0-16.8) |
| HR (95% CI)^*^ | Reference | 1.16 (0.99-1.35) |
| HR (95% CI)^**^ | Reference | 1.01 (0.85-1.20) |
| HR (95% CI)^***^ | Reference | 0.87 (0.73-1.04) |
| HF hospitalization or cardiovascular death | | |
| N (%) | 488 (17.2) | 232 (20.6) |
| Event rate per 100 person-years (95% CI) | 12.5 (11.4-13.7) | 14.5 (12.8-16.5) |
| HR (95% CI)^*^ | Reference | 1.16 (0.99-1.35) |
| HR (95% CI)^**^ | Reference | 1.01 (0.85-1.20) |
| HR (95% CI)^***^ | Reference | 0.86 (0.73-1.03) |
| HF hospitalization | | |
| N (%) | 287 (10.1) | 152 (13.5) |
| Event rate per 100 person-years (95% CI) | 7.4 (6.5-8.3) | 9.5 (8.1-11.1) |
| HR (95% CI)^*^ | Reference | 1.28 (1.05-1.56) |
| HR (95% CI)^**^ | Reference | 1.05 (0.84-1.30) |
| HR (95% CI)^***^ | Reference | 0.90 (0.72-1.12) |
| Cardiovascular death | | |
| N (%) | 291 (10.3) | 124 (11.0) |
| Event rate per 100 person-years (95% CI) | 7.1 (6.3-8.0) | 7.3 (6.1-8.7) |
| HR (95% CI)^*^ | Reference | 1.01 (0.82-1.25) |
| HR (95% CI)^**^ | Reference | 0.91 (0.72-1.15) |
| HR (95% CI)^***^ | Reference | 0.77 (0.61-0.97) |
| All-cause death | | |
| N (%) | 347 (12.2) | 149 (13.2) |
| Event rate per 100 person-years (95% CI) | 8.5 (7.6-9.4) | 8.7 (7.4-10.3) |
| HR (95% CI)^*^ | Reference | 1.03 (0.85-1.24) |
| HR (95% CI)^**^ | Reference | 0.86 (0.70-1.07) |
| HR (95% CI)^***^ | Reference | 0.73 (0.59-0.91) |
| Stroke | | |
| N (%) | 54 (1.9) | 24 (2.1) |
| Event rate per 100 person-years (95% CI) | 1.3 (1.0-1.7) | 1.4 (1.0-2.1) |
| HR (95% CI)^*^ | Reference | 1.06 (0.65-1.72) |
| HR (95% CI)^**^ | Reference | 0.97 (0.56-1.65) |
| HR (95% CI)^***^ | Reference | 0.90 (0.52-1.55) |

*CI, confidence interval; HR, hazard ratio.*

**Cause-specific Cox regression models stratified according to diabetes mellitus status and adjusted for a history of HF hospitalization and randomized treatment allocation. No adjustment for HF hospitalization in model for all-cause death.*

***Cause-specific Cox regression models stratified according to diabetes mellitus status and adjusted for a history of HF hospitalization, randomized treatment allocation, age, sex, geographical region, heart rate, systolic blood pressure, body mass index, HF aetiology, left ventricular ejection fraction, NYHA functional class, estimated glomerular filtration rate, hypertension, type 2 diabetes, chronic obstructive pulmonary disease, and prior stroke/transient ischemic attack. No adjustment for HF hospitalization in model for all-cause death.*

****Fully adjusted cause-specific Cox regression models, including adjustment for N-terminal pro-B-type natriuretic peptide.*

**eTable 4. Time to first event according to type of AF**

|  | No AF  N=2,834 | Paroxysmal AF  N=703 | Persistent/permanent AF  N=1,207 |
| --- | --- | --- | --- |
| Worsening HF event or cardiovascular death | | | |
| N (%) | 494 (17.4) | 139 (19.8) | 255 (21.1) |
| Event rate per 100 person-years (95% CI) | 12.7 (11.6-13.8) | 14.4 (12.2-17.0) | 15.0 (13.3-16.9) |
| HR (95% CI)^*^ | Reference | 1.16 (0.96-1.40) | 1.18 (1.02-1.38) |
| HR (95% CI)^**^ | Reference | 1.07 (0.88-1.31) | 1.01 (0.86-1.19) |
| HR (95% CI)^***^ | Reference | 0.98 (0.80-1.19) | 0.88 (0.75-1.04) |
| HF hospitalization or cardiovascular death | | | |
| N (%) | 488 (17.2) | 138 (19.6) | 251 (20.8) |
| Event rate per 100 person-years (95% CI) | 12.5 (11.4-13.7) | 14.3 (12.1-16.9) | 14.7 (13.0-16.6) |
| HR (95% CI)^*^ | Reference | 1.17 (0.97-1.41) | 1.18 (1.01-1.37) |
| HR (95% CI)^**^ | Reference | 1.08 (0.89-1.32) | 1.01 (0.85-1.19) |
| HR (95% CI)^***^ | Reference | 0.99 (0.81-1.20) | 0.87 (0.74-1.03) |
| HF hospitalization | | | |
| N (%) | 287 (10.1) | 91 (12.9) | 171 (14.2) |
| Event rate per 100 person-years (95% CI) | 7.4 (6.5-8.3) | 9.4 (7.7-11.6) | 10.0 (8.6-11.6) |
| HR (95% CI)^*^ | Reference | 1.31 (1.04-1.66) | 1.36 (1.12-1.64) |
| HR (95% CI)^**^ | Reference | 1.13 (0.88-1.44) | 1.09 (0.88-1.34) |
| HR (95% CI)^***^ | Reference | 1.03 (0.80-1.31) | 0.95 (0.77-1.17) |
| Cardiovascular death | | | |
| N (%) | 291 (10.3) | 76 (10.8) | 133 (11.0) |
| Event rate per 100 person-years (95% CI) | 7.1 (6.3-8.0) | 7.4 (5.9-9.3) | 7.3 (6.1-8.6) |
| HR (95% CI)^*^ | Reference | 1.06 (0.83-1.37) | 1.02 (0.83-1.25) |
| HR (95% CI)^**^ | Reference | 1.03 (0.80-1.34) | 0.91 (0.73-1.13) |
| HR (95% CI)^***^ | Reference | 0.93 (0.71-1.21) | 0.77 (0.62-0.97) |
| All-cause death | | | |
| N (%) | 347 (12.2) | 101 (14.4) | 157 (13.0) |
| Event rate per 100 person-years (95% CI) | 8.5 (7.6-9.4) | 9.9 (8.1-12.0) | 8.6 (7.3-10.0) |
| HR (95% CI)^*^ | Reference | 1.19 (0.95-1.48) | 1.01 (0.84-1.22) |
| HR (95% CI)^**^ | Reference | 1.10 (0.87-1.38) | 0.87 (0.71-1.06) |
| HR (95% CI)^***^ | Reference | 1.00 (0.79-1.26) | 0.74 (0.60-0.91) |
| Stroke | | | |
| N (%) | 54 (1.9) | 8 (1.1) | 26 (2.2) |
| Event rate per 100 person-years (95% CI) | 1.3 (1.0-1.7) | 0.8 (0.4-1.6) | 1.4 (1.0-2.1) |
| HR (95% CI)^*^ | Reference | 0.61 (0.29-1.28) | 1.08 (0.67-1.72) |
| HR (95% CI)^**^ | Reference | 0.63 (0.29-1.34) | 0.95 (0.57-1.59) |
| HR (95% CI)^***^ | Reference | 0.61 (0.29-1.31) | 0.90 (0.54-1.52) |

*CI, confidence interval; HR, hazard ratio.*

**Cause-specific Cox regression models stratified according to diabetes mellitus status and adjusted for a history of HF hospitalization and randomized treatment allocation. No adjustment for HF hospitalization in model for all-cause death.*

***Cause-specific Cox regression models stratified according to diabetes mellitus status and adjusted for a history of HF hospitalization, randomized treatment allocation, age, sex, geographical region, heart rate, systolic blood pressure, body mass index, HF aetiology, left ventricular ejection fraction, NYHA functional class, estimated glomerular filtration rate, hypertension, type 2 diabetes, chronic obstructive pulmonary disease, and prior stroke/transient ischemic attack. No adjustment for HF hospitalization in model for all-cause death.*

****Fully adjusted cause-specific Cox regression models, including adjustment for N-terminal pro-B-type natriuretic peptide.*

**eTable 5. Effects of dapagliflozin compared with placebo on clinical events: No AF (history or baseline ECG) versus AF on ECG (irrespective of history)**

| Outcome | No AF  N=2,834 | | AF on ECG  N=1,128 | | P-value for interaction |
| --- | --- | --- | --- | --- | --- |
|  | Placebo  N=1,415 | Dapagliflozin  N=1,419 | Placebo  N=559 | Dapagliflozin  N=569 |  |
| Worsening HF event or cardiovascular death | | | | | 0.52 |
| N (%) | 281 (19.9) | 213 (15.0) | 126 (22.5) | 109 (19.2) |  |
| Event rate per 100 person-years (95% CI) | 14.7 (13.0-16.5) | 10.8 (9.4-12.3) | 16.2 (13.6-19.3) | 13.4 (11.1-16.1) |  |
| HR (95% CI) | 0.74 (0.62-0.88) | | 0.82 (0.63-1.06) | |  |
| HF hospitalization or cardiovascular death | | | | | 0.65 |
| N (%) | 276 (19.5) | 212 (14.9) | 125 (22.4) | 107 (18.8) |  |
| Event rate per 100 person-years (95% CI) | 14.3 (12.7-16.1) | 10.7 (9.4-12.3) | 16.0 (13.5-19.1) | 13.1 (10.8-15.8) |  |
| HR (95% CI) | 0.75 (0.63-0.89) | | 0.81 (0.63-1.05) | |  |
| HF hospitalization | | | | | 0.46 |
| N (%) | 170 (12.0) | 117 (8.2) | 83 (14.8) | 69 (12.1) |  |
| Event rate per 100 person-years (95% CI) | 8.8 (7.6-10.3) | 5.9 (4.9-7.1) | 10.6 (8.6-13.2) | 8.4 (6.7-10.7) |  |
| HR (95% CI) | 0.67 (0.53-0.85) | | 0.78 (0.57-1.08) | |  |
| Cardiovascular death | | | | | 0.14 |
| N (%) | 161 (11.4) | 130 (9.2) | 58 (10.4) | 66 (11.6) |  |
| Event rate per 100 person-years (95% CI) | 7.9 (6.8-9.2) | 6.3 (5.3-7.5) | 6.9 (5.3-8.9) | 7.6 (6.0-9.7) |  |
| HR (95% CI) | 0.80 (0.64-1.01) | | 1.10 (0.77-1.57) | |  |
| All-cause death | | | | | 0.07 |
| N (%) | 196 (13.9) | 151 (10.6) | 70 (12.5) | 79 (13.9) |  |
| Event rate per 100 person-years (95% CI) | 9.6 (8.4-11.1) | 7.3 (6.3-8.6) | 8.3 (6.6-10.5) | 9.1 (7.3-11.4) |  |
| HR (95% CI) | 0.76 (0.62-0.94) | | 1.09 (0.79-1.51) | |  |
| Recurrent HF hospitalization or cardiovascular death | | | | | 0.17 |
| No. of events | 408 | 294 | 183 | 173 |  |
| RR (95% CI) | 0.71 (0.58-0.87) | | 0.91 (0.68-1.20) | |  |
| KCCQ-TSS | | | | | |
| Change in KCCQ-TSS score at 8 months | 3.3 (2.2-4.4) | 5.8 (4.8-6.9) | 4.8 (3.0-6.5) | 7.5 (5.7-9.3) | 0.26 |
| >5-point improvement in KCCQ-TSS at 8 months | | | | | 0.95 |
| Proportion of patients | 52.2 | 59.1 | 50.8 | 57.4 |  |
| OR (95% CI) | 1.14 (1.06-1.24) | | 1.15 (1.02-1.31) | |  |
| >5-point decrease in KCCQ-TSS at 8 months | | | | | 0.32 |
| Proportion of patients | 31.8 | 25.5 | 33.7 | 24.6 |  |
| OR (95% CI) | 0.86 (0.79-0.94) | | 0.80 (0.69-0.91) | |  |

*CI, confidence interval; HF, heart failure; HR, hazard ratio; KCCQ, Kansas City Cardiomyopathy Questionnaire; TSS, total symptom score; OR, odds ratio; RR, rate ratio.*

**eTable 6. Adverse events of dapagliflozin compared with placebo: No AF (history or baseline ECG) versus AF on ECG (irrespective of history)**

| Adverse event | No AF  N=2,829 | | AF on ECG  N=1,127 | | P-value for interaction |
| --- | --- | --- | --- | --- | --- |
|  | Placebo  N=1,413 | Dapagliflozin  N=1,416 | Placebo  N=558 | Dapagliflozin  N=569 |  |
| Discontinuation of study drug for any reason | 144 (10.1) | 144 (10.2) | 61 (10.9) | 56 (9.8) | 0.62 |
| Discontinuation of study drug due to adverse event | 64 (4.5) | 60 (4.2) | 26 (4.7) | 26 (4.6) | 0.88 |
| Volume depletion | 83 (5.9) | 101 (7.1) | 36 (6.5) | 43 (7.6) | 0.89 |
| Renal adverse event | 100 (7.1) | 77 (5.4) | 31 (5.6) | 46 (8.1) | 0.02 |
| Fracture | 27 (1.9) | 26 (1.8) | 14 (2.5) | 15 (2.6) | 0.84 |
| Amputation | 6 (0.4) | 10 (0.7) | 3 (0.5) | 3 (0.5) | 0.58 |
| Major hypoglycaemia | 1 (0.1) | 2 (0.1) | 2 (0.4) | 2 (0.4) | 0.65 |
| Diabetic ketoacidosis | 0 (0.0) | 1 (0.1) | 0 (0.0) | 2 (0.4) | N/A |

*A total of six randomized patients were excluded from the safety analysis, as these were performed in patients who had undergone randomization and received at least one dose of dapagliflozin or placebo.*

**eTable 7. Effects of dapagliflozin compared with placebo on clinical events according to type of AF**

| Outcome | No AF  N=2,834 | | Paroxysmal AF  N=703 | | Persistent/permanent AF  N=1,207 | | P-value for  interaction |
| --- | --- | --- | --- | --- | --- | --- | --- |
|  | Placebo  N=1,415 | Dapagliflozin  N=1,419 | Placebo  N=349 | Dapagliflozin  N=354 | Placebo  N=607 | Dapagliflozin  N=600 |  |
| Worsening HF event or cardiovascular death | | | | | | | 0.77 |
| N (%) | 281 (19.9) | 213 (15.0) | 75 (21.5) | 64 (18.1) | 146 (24.1) | 109 (18.2) |  |
| Event rate per 100 person-years (95% CI) | 14.7 (13.0-16.5) | 10.8 (9.4-12.3) | 16.0 (12.7-20.0) | 13.0 (10.2-16.6) | 17.4 (14.8-20.5) | 12.6 (10.5-15.2) |  |
| HR (95% CI) | 0.74 (0.62-0.88) | | 0.83 (0.59-1.15) | | 0.72 (0.56-0.92) | |  |
| HF hospitalization or cardiovascular death | | | | | | | 0.81 |
| N (%) | 276 (19.5) | 212 (14.9) | 75 (21.5) | 63 (17.8) | 144 (23.7) | 107 (17.8) |  |
| Event rate per 100 person-years (95% CI) | 14.3 (12.7-16.1) | 10.7 (9.4-12.3) | 16.0 (12.7-20.0) | 12.8 (10.0-16.3) | 17.1 (14.5-20.2) | 12.3 (10.2-14.9) |  |
| HR (95% CI) | 0.75 (0.63-0.89) | | 0.81 (0.58-1.14) | | 0.71 (0.56-0.92) | |  |
| HF hospitalization | | | | | | | 0.28 |
| N (%) | 170 (12.0) | 117 (8.2) | 46 (13.2) | 45 (12.7) | 102 (16.8) | 69 (11.5) |  |
| Event rate per 100 person-years (95% CI) | 8.8 (7.6-10.3) | 5.9 (4.9-7.1) | 9.8 (7.3-13.1) | 9.1 (6.8-12.2) | 12.1 (10.0-14.7) | 8.0 (6.3-10.1) |  |
| HR (95% CI) | 0.67 (0.53-0.85) | | 0.94 (0.63-1.42) | | 0.65 (0.48-0.88) | |  |
| Cardiovascular death | | | | | | | 0.93 |
| N (%) | 161 (11.4) | 130 (9.2) | 41 (11.7) | 35 (9.9) | 71 (11.7) | 62 (10.3) |  |
| Event rate per 100 person-years (95% CI) | 7.9 (6.8-9.2) | 6.3 (5.3-7.5) | 8.1 (6.0-11.0) | 6.8 (4.9-9.4) | 7.8 (6.2-9.8) | 6.8 (5.3-8.7) |  |
| HR (95% CI) | 0.80 (0.64-1.01) | | 0.85 (0.54-1.33) | | 0.86 (0.61-1.21) | |  |
| All-cause death |  |  |  |  |  |  | 0.41 |
| N (%) | 196 (13.9) | 151 (10.6) | 50 (14.3) | 51 (14.4) | 83 (13.7) | 74 (12.3) |  |
| Event rate per 100 person-years (95% CI) | 9.6 (8.4-11.1) | 7.3 (6.3-8.6) | 9.9 (7.5-13.0) | 9.9 (7.5-13.0) | 9.1 (7.3-11.3) | 8.1 (6.4-10.1) |  |
| HR (95% CI) | 0.76 (0.62-0.94) | | 1.01 (0.69-1.50) | | 0.88 (0.64-1.20) | |  |
| Recurrent HF hospitalization or cardiovascular death | | | | | | | 0.65 |
| No. of events | 408 | 294 | 115 | 100 | 219 | 173 |  |
| RR (95% CI) | 0.71 (0.58-0.87) | | 0.87 (0.60-1.26) | | 0.77 (0.58-1.02) | |  |
| KCCQ-TSS | | | | | | | |
| Change in KCCQ-TSS score at 8 months | 3.3 (2.2-4.4) | 5.8 (4.8-6.9) | 1.9 (-0.3 to 4.2) | 6.1 (4.0-8.2) | 4.1 (2.3-5.8) | 6.8 (5.1-8.4) | 0.69 |
| >5-point improvement in KCCQ-TSS at 8 months | | | | | | | 0.82 |
| Proportion of patients | 52.2 | 59.1 | 47.0 | 58.2 | 50.3 | 56.4 |  |
| OR (95% CI) | 1.14 (1.06-1.24) | | 1.24 (1.06-1.44) | | 1.14 (1.00-1.29) | |  |
| >5-point decrease in KCCQ-TSS at 8 months | | | | | | | 0.86 |
| Proportion of patients | 31.8 | 25.5 | 34.5 | 24.4 | 34.5 | 25.4 |  |
| OR (95% CI) | 0.86 (0.79-0.94) | | 0.79 (0.67-0.94) | | 0.80 (0.70-0.91) | |  |

*CI, confidence interval; HF, heart failure; HR, hazard ratio; KCCQ, Kansas City Cardiomyopathy Questionnaire; TSS, total symptom score; OR, odds ratio; RR, rate ratio.*

**eTable 8. Adverse events of dapagliflozin compared with placebo according to type of AF**

| Adverse event | No AF  N=2,829 | | Paroxysmal AF  N=701 | | Persistent/permanent AF  N=1,206 | | P-value for interaction |
| --- | --- | --- | --- | --- | --- | --- | --- |
|  | Placebo  N=1,413 | Dapagliflozin  N=1,416 | Placebo  N=348 | Dapagliflozin  N=353 | Placebo  N=607 | Dapagliflozin  N=599 |  |
| Discontinuation of study drug for any reason | 144 (10.1) | 144 (10.2) | 45 (12.9) | 45 (12.7) | 69 (11.4) | 60 (10.0) | 0.82 |
| Discontinuation of study drug due to adverse event | 64 (4.5) | 60 (4.2) | 21 (6.0) | 25 (7.1) | 31 (5.1) | 26 (4.3) | 0.69 |
| Volume depletion | 83 (5.9) | 101 (7.1) | 36 (10.3) | 30 (8.5) | 43 (7.1) | 47 (7.8) | 0.37 |
| Renal adverse event | 100 (7.1) | 77 (5.4) | 33 (9.5) | 31 (8.8) | 37 (6.1) | 45 (7.5) | 0.19 |
| Fracture | 27 (1.9) | 26 (1.8) | 13 (3.7) | 8 (2.3) | 10 (1.6) | 15 (2.5) | 0.31 |
| Amputation | 6 (0.4) | 10 (0.7) | 2 (0.6) | 0 (0.0) | 4 (0.7) | 3 (0.5) | 0.70 |
| Major hypoglycaemia | 1 (0.1) | 2 (0.1) | 1 (0.3) | 0 (0.0) | 2 (0.3) | 2 (0.3) | 0.91 |
| Diabetic ketoacidosis | 0 (0.0) | 1 (0.1) | 0 (0.0) | 1 (0.3) | 0 (0.0) | 1 (0.2) | N/A |

*A total of eight randomized patients were excluded from the safety analysis, as these were performed in patients who had undergone randomization and received at least one dose of dapagliflozin or placebo.*

**eTable 9. Characteristics of new-onset AF status in patients without AF (history or baseline ECG)**

| **Characteristics** | **New-onset AF**  **N=123** |
| --- | --- |
| Documented on ECG^*^ |  |
| No | 15 (12.3) |
| Yes | 107 (87.7) |
| Symptomatic^**^ |  |
| No | 60 (49.6) |
| Yes | 61 (50.4) |
| Type |  |
| Paroxysmal | 71 (57.7) |
| Persistent | 42 (34.1) |
| Permanent | 10 (8.1) |
| Any treatment^*^ |  |
| No | 29 (23.8) |
| Yes | 93 (76.2) |
| Rate control therapy |  |
| No | 85 (69.7) |
| Yes | 37 (30.3) |
| Antiarrhythmic drugs^*^ |  |
| No | 84 (68.9) |
| Yes | 38 (31.1) |
| Invasive antiarrhythmic therapy^*^*^#^* |  |
| No | 116 (95.1) |
| Yes | 6 (4.9) |
| Electric cardioversion^*^ |  |
| No | 103 (84.4) |
| Yes | 19 (15.6) |
| Anticoagulation therapy^*^ |  |
| No | 48 (39.3) |
| Yes | 74 (60.7) |
| Antiplatelet therapy^*^ |  |
| No | 111 (91.0) |
| Yes | 11 (9.0) |

*^*^Data missing for 1 patient.*

*^**^Data missing for 2 patients.*

*^#^Percutaneous/surgical ablation or pacemaker insertion to facilitate rhythm control.*

**eTable 10. Baseline characteristics by new-onset AF status in patients without AF (history or baseline ECG)**

|  | No new-onset AF  N=2,711 | New-onset AF  N=123 | P-value |
| --- | --- | --- | --- |
| Age (years), mean (SD) | 64.2 (11.2) | 67.8 (10.9) | <0.001 |
| Sex, N (%) |  |  | 0.40 |
| Female | 686 (25.3) | 27 (22.0) |  |
| Male | 2,025 (74.7) | 96 (78.0) |  |
| Race, N (%) |  |  | 0.003 |
| Asian | 788 (29.1) | 18 (14.6) |  |
| Black | 151 (5.6) | 5 (4.1) |  |
| White | 1,727 (63.7) | 98 (79.7) |  |
| Other | 45 (1.7) | 2 (1.6) |  |
| Geographic region, N (%) |  |  | <0.001 |
| Asia/Pacific | 773 (28.5) | 18 (14.6) |  |
| Europe | 1,014 (37.4) | 71 (57.7) |  |
| North America | 373 (13.8) | 18 (14.6) |  |
| South America | 551 (20.3) | 16 (13.0) |  |
| Physiologic measures |  |  |  |
| Systolic blood pressure (mmHg), mean (SD) | 122.0 (16.8) | 120.7 (15.4) | 0.38 |
| Heart rate (bpm), mean (SD) | 70.8 (10.7) | 68.8 (11.3) | 0.06 |
| BMI (kg/m^2^), mean (SD) | 27.9 (5.9) | 28.6 (5.2) | 0.06 |
| Creatinine (μmol/L), mean (SD) | 101.5 (30.4) | 105.8 (34.5) | 0.17 |
| Glycated haemoglobin, mean (SD) | 6.1 (5.7-7.0) | 5.9 (5.5-6.9) | 0.08 |
| eGFR (mL/min/1.73m^2^), mean (SD) | 68.7 (20.1) | 64.7 (19.4) | 0.03 |
| eGFR (mL/min/1.73m^2^), N (%) |  |  | 0.21 |
| < 60 | 951 (35.1) | 50 (40.7) |  |
| > 60 | 1,758 (64.9) | 73 (59.3) |  |
| NT-proBNP, median (IQR) | 1,221 (737-2,293) | 1,803 (1,048-2,923) | <0.001 |
| Main cause of HF, N (%) |  |  | 0.29 |
| Ischemic | 1,641 (60.5) | 82 (66.7) |  |
| Non-ischemic | 855 (31.5) | 35 (28.5) |  |
| Unknown | 215 (7.9) | 6 (4.9) |  |
| Duration of HF, N (%) |  |  | <0.001 |
| 0-3 months | 94 (3.5) | 0 (0.0) |  |
| 3-6 months | 252 (9.3) | 8 (6.5) |  |
| 6-12 months | 375 (13.8) | 1 (0.8) |  |
| 1-2 years | 436 (16.1) | 14 (11.4) |  |
| 2-5 years | 616 (22.7) | 36 (29.3) |  |
| >5 years | 938 (34.6) | 64 (52.0) |  |
| LVEF, mean (SD) | 30.5 (6.8) | 30.8 (6.4) | 0.68 |
| NYHA class, N (%) |  |  | 0.57 |
| II | 1,909 (70.4) | 86 (69.9) |  |
| III | 779 (28.7) | 37 (30.1) |  |
| IV | 23 (0.8) | 0 (0.0) |  |
| KCCQ-TSS, mean (SD) | 75.0 (21.5) | 71.3 (21.7) | 0.07 |
| Medical history, N (%) |  |  |  |
| Hospitalization for HF | 1,259 (46.4) | 53 (43.1) | 0.47 |
| Hypertension | 1,899 (70.0) | 102 (82.9) | 0.002 |
| Type 2 diabetes | 1,250 (46.1) | 58 (47.2) | 0.82 |
| Chronic obstructive pulmonary disease | 288 (10.6) | 19 (15.4) | 0.09 |
| Previous MI | 1,318 (48.6) | 73 (59.3) | 0.02 |
| Previous stroke or TIA | 268 (9.9) | 17 (13.8) | 0.16 |
| Peripheral artery disease | 377 (13.9) | 25 (20.3) | 0.046 |
| Treatment, N (%) |  |  |  |
| ACEI/ARB | 2,301 (84.9) | 99 (80.5) | 0.19 |
| ARNI | 273 (10.1) | 14 (11.4) | 0.64 |
| ACEI/ARB/ARNI | 2,562 (94.5) | 113 (91.9) | 0.21 |
| Beta-blocker | 2,621 (96.7) | 118 (95.9) | 0.61 |
| MRA | 1,973 (72.8) | 93 (75.6) | 0.49 |
| Ivabradine | 190 (7.0) | 12 (9.8) | 0.25 |
| Digoxin | 312 (11.5) | 14 (11.4) | 0.97 |
| Amiodarone | 204 (7.5) | 11 (8.9) | 0.56 |
| Class I antiarrythmic drugs | 5 (0.2) | 1 (0.8) | 0.23 |
| Sotalol | 16 (0.6) | 1 (0.8) | 0.53 |
| Oral anticoagulant* | 355 (13.1) | 17 (13.8) | 0.82 |
| Antiplatelet** | 1,920 (70.8) | 98 (79.7) | 0.03 |
| CRT-P/CRT-D | 166 (6.1) | 12 (9.8) | 0.10 |
| ICD/CRT-D | 631 (23.3) | 39 (31.7) | 0.03 |
| CHA_2_DS_2_-VASc score, mean (SD) | 3.9 (1.6) | 4.4 (1.5) | <0.001 |
| CHA_2_DS_2_-VASc score >2, N (%) | 2,559 (94.4) | 121 (98.4) | 0.06 |

*ACE angiotensin-converting enzyme; ARB, angiotensin receptor blocker; ARNI, angiotensin receptor neprilysin inhibitor; BMI, body mass index; CRT-D, cardiac resynchronization therapy–defibrillator; CHA_2_DS_2_-VASc, congestive heart failure, hypertension, age >75 years; diabetes mellitus, prior stroke or transient ischemic attack or thromboembolism, vascular disease, age 65–74 years, sex category; CRT-P, cardiac resynchronization therapy–pacemaker; eGFR, estimated glomerular filtration rate; HF, heart failure; ICD, implantable cardioverter-defibrillator; IQR, interquartile range; KCCQ-TTS, Kansas City Cardiomyopathy Questionnaire Total Symptom Score; MI, myocardial infarction; NT-proBNP, N-terminal pro-B-type natriuretic peptide; NYHA, New York Heart Association; TIA, transient ischemic attack.*

**Vitamin K antagonists (warfarin/coumadin) and “direct” oral anticoagulants (dabigatran, rivaroxaban, apixaban, and edoxaban).*

***Aspirin, ADP-receptor inhibitors (clopidogrel, ticagrelor, prasugrel), and adenosine reuptake inhibitors (dipyridamole).*
